# Constraints from Comets on the Formation and Volatile Acquisition of the Planets and Satellites

**DOI:** 10.1007/s11214-015-0161-z

**Published:** 2015-05-21

**Authors:** K.E. Mandt, O. Mousis, B. Marty, T. Cavalié, W. Harris, P. Hartogh, K. Willacy

**Affiliations:** 1 Southwest Research Institute, San Antonio, TX, USA; 2 Aix Marseille Université, CNRS, LAM (Laboratoire d’Astrophysique de Marseille) UMR 7326, 13388, Marseille, France; 3 CRPG-CNRS, Nancy-Université, Vandoeuvre-lès-Nancy, France; 4 Max Planck Institute for Solar System Research, Göttingen, Germany; 5 University of Arizona, Tucson, AZ, USA; 6 Jet Propulsion Laboratory, Pasadena, CA, USA

**Keywords:** Solar system formation, Comets, Atmospheres, Giant planets, Terrestrial planets, Moon formation

## Abstract

Comets play a dual role in understanding the formation and evolution of the solar system. First, the composition of comets provides information about the origin of the giant planets and their moons because comets formed early and their composition is not expected to have evolved significantly since formation. They, therefore serve as a record of conditions during the early stages of solar system formation. Once comets had formed, their orbits were perturbed allowing them to travel into the inner solar system and impact the planets. In this way they contributed to the volatile inventory of planetary atmospheres. We review here how knowledge of comet composition up to the time of the Rosetta mission has contributed to understanding the formation processes of the giant planets, their moons and small icy bodies in the solar system. We also discuss how comets contributed to the volatile inventories of the giant and terrestrial planets.

## 1 Introduction

Understanding the formation and evolution of the solar system requires evaluation of measurements that have been made in various solar system bodies that include the Sun, terrestrial planet mantles and atmospheres, giant planet atmospheres, giant planet satellites, meteorites and comets. These measurements are compared to models for solar system formation and evolution in an iterative manner where new measurements improve models and models provide guidelines for future measurements that are needed.

Measurements of cometary composition provide constraints in two areas. First, cometary composition provides a tracer of chemical conditions during solar system formation. Comets are small bodies that formed from ices and dust in the protosolar nebula (PSN) and are presumed to have evolved very little since their initial formation. Their composition is representative of conditions in the region in which they formed. After formation, the orbits of many comets were perturbed by migration of the giant planets causing them to impact the planets and contribute to planetary volatile inventories. Our goal is to review current state of knowledge—including some early Rosetta mission results—on the role of comets in understanding the formation and evolution of other solar system bodies.

In Sect. 2 we outline the current understanding of the formation of the solar system and the relevant composition of the PSN. We then discuss what is known about the formation of the giant planets in Sect. 3 and their satellites in Sect. 4. The role of comets in producing the volatile inventories of the terrestrial planets is discussed in Sect. 5, and Pluto and Kuiper Belt objects are covered in Sect. 6. We summarize the current state of knowledge, what we expect to learn from future measurements and what future measurements are needed in Sect. 7.

## 2 Formation of the Solar System

In order to evaluate how comets contribute to understanding the formation and evolution of other bodies in the solar system, we must first understand how the solar system formed and the composition of the PSN during the time of formation of comets and other solar system bodies. There are two types of models used to study the formation and evolution of the solar system: chemical models that describe the composition and chemistry in the PSN (for a more detailed description see the review by Willacy et al. 2015, this issue), and dynamical models that evaluate the physical processes determining where objects formed and how they ended up where they are today (for a more detailed description see the review by Dones et al. 2015, this issue).

Measurements made in various solar system bodies are used to determine the bulk composition of the PSN and the variability of its composition with distance from the young Sun. This “taxonomy” of solar system measurements can provide a basis for investigating processes within the PSN and processes that led to the evolution of solar system bodies after their formation. The most valuable measurements include noble gas abundances, noble gas isotope ratios, relative abundances of hydrogen, nitrogen, carbon and oxygen and their stable isotope ratios.

### 2.1 The Formation Processes of the Solar System and Conditions in the Solar Nebula

#### 2.1.1 Initial Stages and Chemistry

**Fig. 1:**
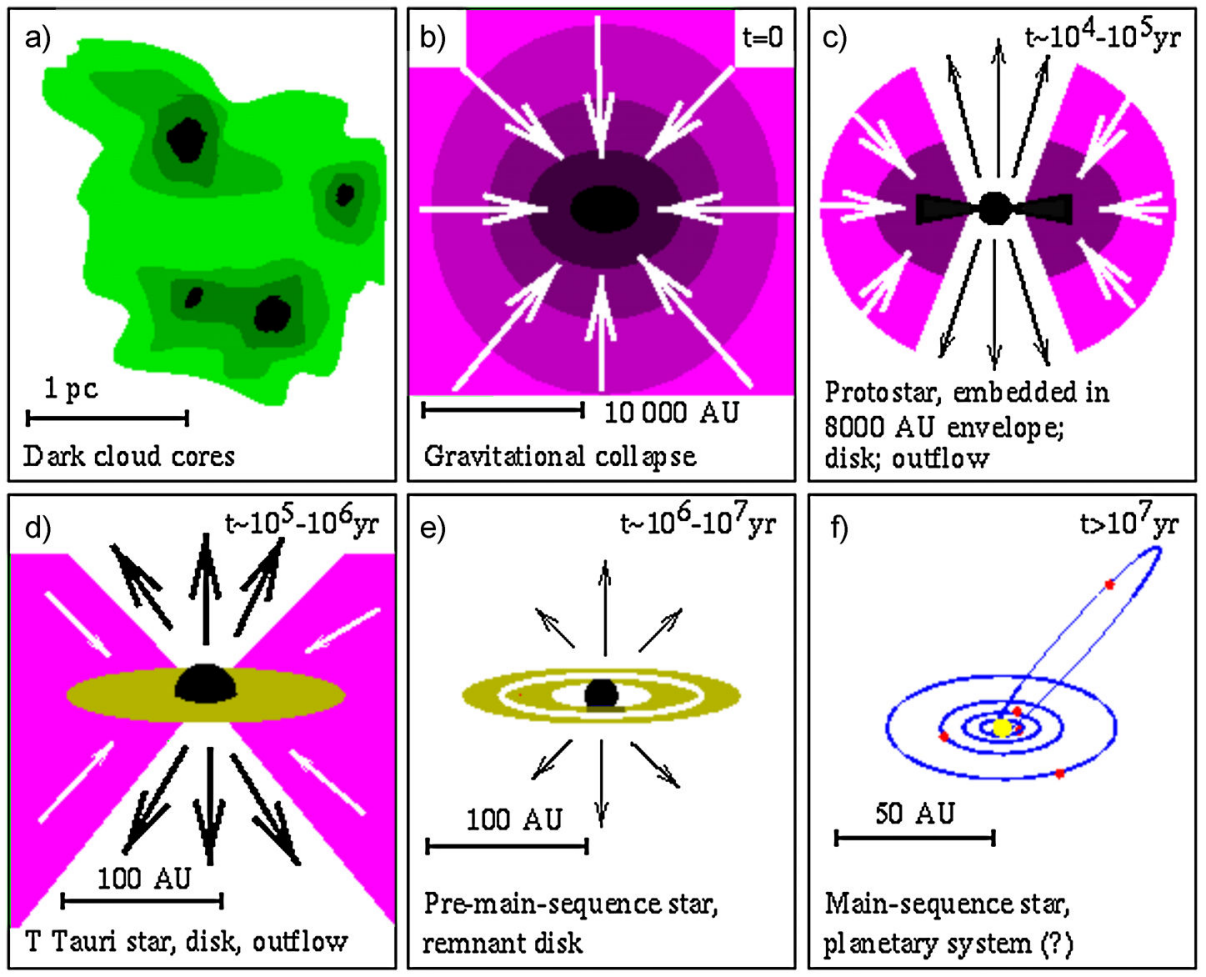
The low mass star formation process (based on Shu et al. 1987). Reprinted from Hogerheijde (1998) with permission

The formation of the solar system began about 4.6 billion years ago with the collapse of a molecular cloud core as illustrated in Fig. 1. Prior to collapse, this core had been evolving over a period of a few million years. Despite very cold temperatures (10 K), a rich chemistry takes place within molecular clouds—almost 200 molecules have currently been identified in interstellar clouds. Additionally, because of the low temperatures, any molecules colliding with dust grains stick to them, forming icy mantles. These ices are also chemically active and more molecules can form by reactions within them. Indeed the most abundant ice species, water, is thought to form mainly by the hydrogenation of oxygen atoms on grain surfaces.

Core collapse occurs from the inside out (Shu 1977), with the protostar forming at the center where the densities are highest (Fig. 1b). Once the star switches on it generates powerful bipolar outflows that begin to clear away the parent cloud. During this stage, infall is still occurring (Fig. 1c) and a disk forms. The infalling material experiences an accretion shock at the surface of the disk, which could alter it chemically, for example by desorption of ices or dissociation of molecules. The importance of the shock in influencing the composition of material that enters the disk is still a matter of debate (e.g. Lunine et al. 1991; Visser et al. 2009).

**Fig. 2:**
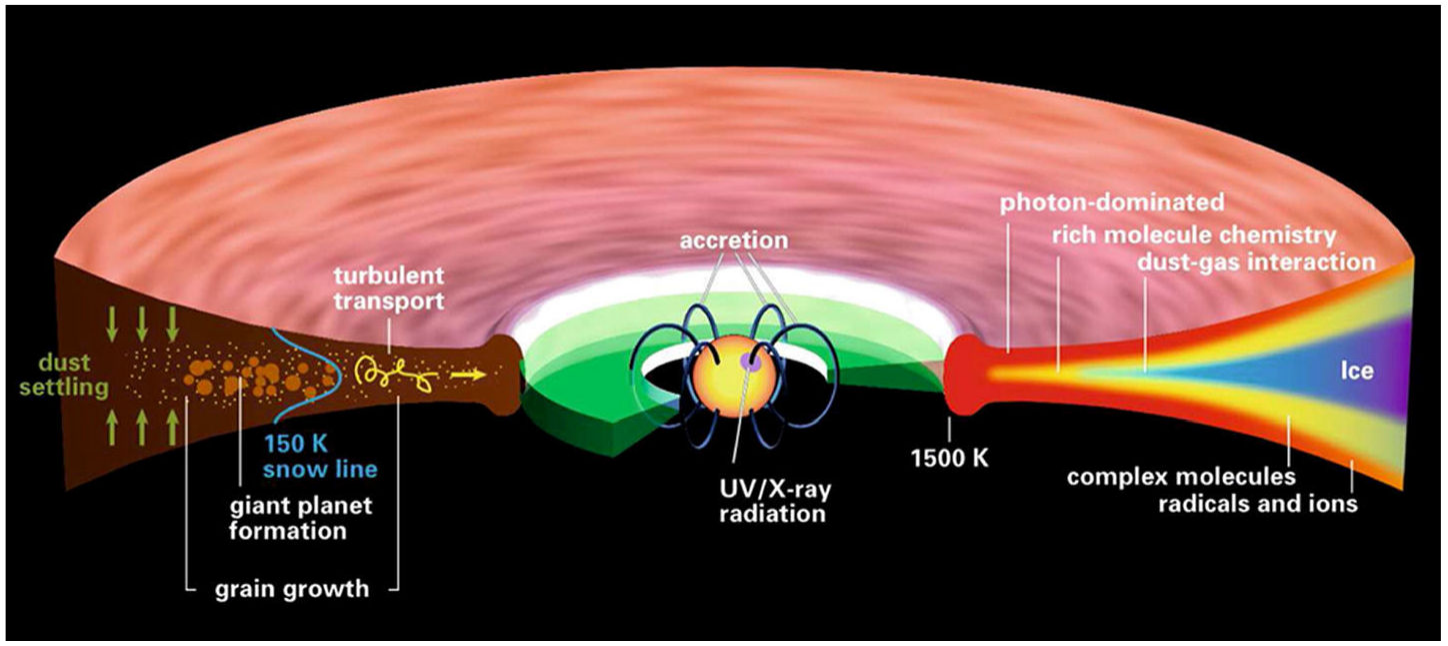
Physical and chemical structure of a 1–5 Myrs protostellar disk around a Sun-like star. Reprinted from Henning and Semenov (2013) with permission. Copyright © 2013 American Chemical Society

In the *T-Tauri* phase (Fig. 1d) the infall is reduced, outflows continue and the disk can now be observed directly. Figure 2 shows the chemical structure of a typical *T-Tauri* disk around a Sun-like star. The wide range of physical conditions (temperature, density and ultraviolet or UV field) generates very different chemistries, from the cold midplane where the majority of molecules are frozen out as ices, to the highly irradiated, photon-dominated surface. Between these two layers is a region where desorption is efficient enough to maintain a population of molecules in the gas and where the UV field is low enough for these molecules to survive. This is the molecular layer that is detected in many observations of protostellar disks. Turbulent mixing is very important at this stage of evolution, with both gas and dust being transported between regions with different physical conditions driving changes in composition (see Willacy et al. 2015, this issue). Grain growth is another crucial process as grains coagulate to form larger particles that decouple from the gas motions and sink towards the midplane. The removal of small grains from the surface layers of the disk changes the opacity and hence the degree to which UV photons can penetrate, thus inducing more chemical changes. All of these chemical changes could be reflected in the composition of the planetesimals, and eventually the planetary bodies that form in the disk (Figs. 1e and 1f).

#### 2.1.2 Formation of Planets, Moons and Small Bodies

The planets, moons and small bodies (including comets) are commonly believed to have formed through the process of accretion, where dust grains form clumps that collide and accumulate into objects known as planetesimals, which are tens of kilometers in size (Goldreich and Ward 1973). Planetesimals that formed closer to the Sun were depleted in volatiles because of high temperatures, while beyond the “snow line” (~4 astronomical units or AU, illustrated in Fig. 2) planetesimals formed with significant amounts of water ice and other volatiles. After formation, the inner planets are thought to have migrated inward from their formation location (e.g. Goldreich and Tremaine 1980). On the other hand, the gas giants, Jupiter, Saturn, Uranus and Neptune, are believed to have formed between 5.5 and 17 AU and then migrated to their present positions of ~5, ~10, ~20 and ~30 AU, respectively (Gomes et al. 2005; Tsiganis et al. 2005; Morbidelli et al. 2005).

There are a variety of methods through which the moons in the solar system formed. Many of the moons of the giant planets are believed to have formed in a subnebula surrounding the planet that they now orbit (e.g. Prinn and Fegley 1989), although some may have formed from building blocks formed earlier in the PSN that migrated into the subnebula (e.g. Mousis et al., 2009a, 2009b; Mandt et al. 2014). Others are thought to be planetesimals that were captured by the planet (e.g. Agnor and Hamilton 2006). Finally, some moons are thought to have formed due to a major collision, including the Earth’s moon (Canup and Asphaug 2001) and Pluto’s moon Charon (Canup 2005). Asteroids and comets are planetesimals left over from the process of forming the planets and the moons.

Based on what is known of their orbital parameters, comets are believed to originate primarily from two regions of the solar system—the Kuiper Belt and scattered disc, which are located 30 to 100 AU from the Sun, and the Oort Cloud which is located beyond 50000 AU. The formation regions of comets have been studied for some time by dynamical models, but many questions remain (see Dones et al. 2015, this issue, for a complete review). A key question for our study is to evaluate whether the comets from the Kuiper belt and Oort Cloud would have compositions that are distinct from each other, or if there is little connection between composition and their current location.

Oort Cloud comets (OCCs) originate from the Oort Cloud and may have formed at a distance of 5–30 AU from the Sun and then scattered to their current location (e.g. Duncan et al. 1987), or they could have been captured from nearby stars in the Sun’s birth cluster (Levison et al. 2010). Jupiter Family Comets (JFCs) are believed to originate in the Kuiper Belt and are suggested to have formed at distances 30–35 AU from the Sun and been scattered outwards (Levison et al. 2008). It would be reasonable to presume that if Kuiper Belt and Oort Cloud objects formed at distinct distances from the Sun with unique temperature and composition conditions in the PSN they would represent two groups of comets with unique compositions (Mumma et al. 2003; Mumma and Charnley 2011). However, another model suggests that all comets formed in a region 15–35 AU from the Sun (Tsiganis et al. 2005; O’Brien et al. 2006) and were scattered into both the Oort Cloud and Kuiper Belt regions by the inward migration of Saturn (Crovisier 2007). In this case comets from both the Kuiper Belt and Oort Cloud would display a variety of compositions with no distinction between the two groups. Therefore, it is unclear at this time whether different comet families can be expected to have different compositions given current uncertainties in their dynamical evolutions.

### 2.2 Defining “Solar Composition”

The first step in understanding formation of the solar system is to constrain the bulk composition of the PSN. The standard reference for this is the chemical composition of the Sun corrected for the evolution of the Sun over the past 4.6 billion years (Ga). Solar chemical composition has been investigated for several decades and knowledge improves regularly thanks to progress made in measurements of the primitive matter of the Solar System, in atomic and molecular data, and in solar atmospheric modeling, allowing better inference of the photospheric abundances. Table 1 represents a list of solar and protosolar abundances of elements derived from the recent compilation of Lodders et al. (2009), which provides the most representative list of abundances of volatiles that potentially existed in the protosolar nebula. In order to compare data to elemental abundances in the early Sun or in the PSN, it is preferable to use the protosolar abundances listed in the table because the Sun’s evolution reduced the abundances of elements heavier than H_2_ by more than 13 % in the photosphere over the last 4.56 Ga (Lodders et al. 2009; Asplund et al. 2009). The conversion of present-day to protosolar abundances is made via a correction of +0.061 logarithmic abundances (dex) for He and +0.053 dex for all other elements except H. The abundances and associated uncertainties are given in dex and converted in ratios relative to H and H_2_.

The isotopes of main volatile elements and their relative proportions are listed in Table 2. These data derive from Lodders et al. (2009) who updated the compilation of isotopes published by Lodders (2003).

### 2.3 Volatiles and Noble Gases in the PSN

Having constrained the bulk elemental composition of the PSN, the next step in understanding the histories of the planets and moons is to constrain the molecular composition and variability of this composition within the PSN. In this way comets are highly valuable as their composition provides a record of the composition of the PSN in the region where they formed (for a more detailed review of comet composition see Cochran et al. 2015, this issue and Bockelée-Morvan et al. 2015, this issue). In addition to comets, meteorites can help to constrain composition within the PSN. Meteorites are presumed to have initially been part of larger planetesimals, of which there are two general categories—differentiated and undifferentiated. Chondritic meteorites are presumed to originate from undifferentiated planetesimals and thus represent the bulk composition of those source planetesimals (Lodders et al. 2009). Chondrites are broken into nine different classes and CI-chondrites demonstrate the closest abundance pattern to solar abundance with the exception of the most volatile elements H, C, N and O (Lodders et al. 2009). Key measurements are noble gas abundances, relative abundances of H, C, N and O and the stable isotope ratios of these elements.

#### 2.3.1 C, H, O and N

The relative abundances of C, H, N and O give a record of volatile history in the PSN and in solar system bodies. The bulk abundance of C, H, N and O in the giant planets is of particular interest as a tracer of their formation processes, and will be discussed further in Sect. 3.

Stable isotope ratios of C, H, N and O are valuable for studying the volatile history of different bodies. They serve as a tracer for temperature conditions and dominant reaction pathways within the PSN as well as for the history of chemical processing of an atmosphere, surface or interior. Furthermore, they can be used to determine the primordial form of volatiles delivered to a solar system body (e.g. Mandt et al. 2014).

Measurements of isotopic ratios for the most abundant elements in cometary and planetary volatiles is still a developing discipline with only a limited number of targets and source species having been studied to date. The observations themselves are challenging, with both high-efficiency and high-spectral resolution needed to detect the weaker isotopic signatures in Earth-based remote sensing. Direct detection from mass spectroscopy can be more effective, but the opportunities in comets are limited to the few JFCs that have been visited by spacecraft (e.g. Balsiger et al. 1995; Eberhardt et al. 1995; Altwegg and Bockelée-Morvan 2003; Altwegg et al. 2015).

The D/H ratio in water depends significantly on temperature of formation while the isotopic ratios of elements heavier than water depend on temperature as well as the mechanism of chemical storage and the current state of the body at the time of release. For molecular radicals in comets, both the source molecule (e.g. HCN for CN) and the mechanism of storage (e.g. ice, clathrate, PAH, silicate grain, etc.) can affect the observed ratio of the same molecular species in different objects formed under nearly identical circumstances.

#### 2.3.2 Water and D/H

The variability of the D/H ratio throughout the solar system provides important clues to solar system formation conditions. Of particular interest is how this ratio varies in water. In prestellar cores the very low temperature conditions cause most volatiles to freeze out and condense onto grains. An upper limit of the gaseous water abundance relative to hydrogen of only 1.3 ppb was recently reported in starless cores (Caselli et al. 2010), while the fraction of water in ice mantles covering dusts grains is about 100 ppm relative to hydrogen (e.g. Whittet and Duley 1991). The formation process of water on ice grains involving chemical reactions favors heavier water isotopologues. Ceccarelli et al. (2005) and Butner et al. (2007) derived D/H ratios in the water of molecular clouds and protostellar envelopes of about 1.0 × 10^−2^ and 1.0 × 10^−3^ respectively. This is an enrichment of 2–3 orders of magnitude relative to the D/H ratio of protosolar hydrogen, 2.1 × 10^−6^, or the local interstellar medium, 1.6 × 10^−6^ (Linsky et al. 2006). These values may be considered as upper and lower limits of the D/H ratio in water in our solar system, taking into account the possibility of isotopic exchange between water and hydrogen.

**Fig. 3:**
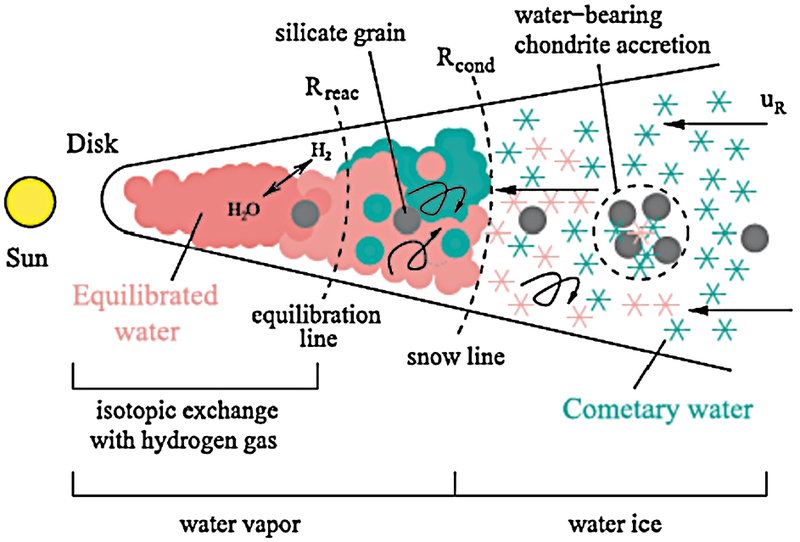
Illustration of the formation of a D/H gradient with heliocentric distance. *Arrows* symbolize motions of gas. Reprinted from Jacquet and Robert (2013) with permission. Copyright © 2013 Elsevier

Figure 3 illustrates how the origin of water in our solar system may be traced back by determination of the water D/H ratio.

In the region of the disk near the sun under pressures in the 10 μbar to 1 mbar range and temperatures between 600 and 1300 K (compare e.g. Yang et al. 2013, Fig. 1), gaseous water equilibrates with hydrogen within a few hundred years. This means that the D/H ratio in water, which was initially highly enriched, will be defractionated to the value of hydrogen. With increasing heliocentric distance, temperature and density drops and the efficiency of the isotopic exchange process decreases and finally stops at an intermediate distance from the sun where water is still present in gaseous form. Here, equilibrated water transported from the inner zone by turbulent diffusion and sublimating water from highly D-enriched ices drifting inwards mix and establish a radial gradient of D/H, becoming fixed beyond the snow line.

Observational constraints on the D/H ratio in water in comets became available for the first time with the Giotto mission. Balsiger et al. (1995) and Eberhardt et al. (1995) derived D/H ratios of about 3.0 × 10^−4^ for 1P/Halley, which originated in the Oort Cloud. This value, about twice as high as the Vienna Standard Mean Ocean Water (VSMOW) of 1.56 × 10^−4^ was confirmed (within error bars) in OCCs by a number of ground-based detections in C/1996 B2 (Hyakutake) (Bockelée-Morvan et al. 1998), C/1995 O1 (Hale-Bopp) (Meier et al. 1998), C/2002 T7 (LINEAR) (Hutsemékers et al. 2008) and 8P/Tuttle (Villanueva et al. 2009). Biver et al. (2006) provided an upper limit for 153P/Ikeya-Zhang of 2.50 × 10^−4^. Waite et al. (2009) added the D/H found in the Enceladus plume, which was in line with D/H of Oort cloud comets.

Simulations of the D/H gradient using upper limits for the ice D/H from the analysis of meteorite samples, managed to verify these observations (e.g. Drouart et al. 1999; Mousis et al. 2000; Horner et al. 2007 and Kavelaars et al. 2011). Therefore, models appear to be compatible with the assumption that OCCs formed in the vicinity of the gas giants. The models indeed show an increase of the water D/H ratio with heliocentric distance. Kavelaars et al. (2011) predicted a higher D/H ratio of at least 5.00 × 10^−4^ for JFCs, believed to have formed at larger heliocentric distances than the OCCs, i.e. beyond Neptune in the Kuiper Belt or scattered disk.

**Fig. 4:**
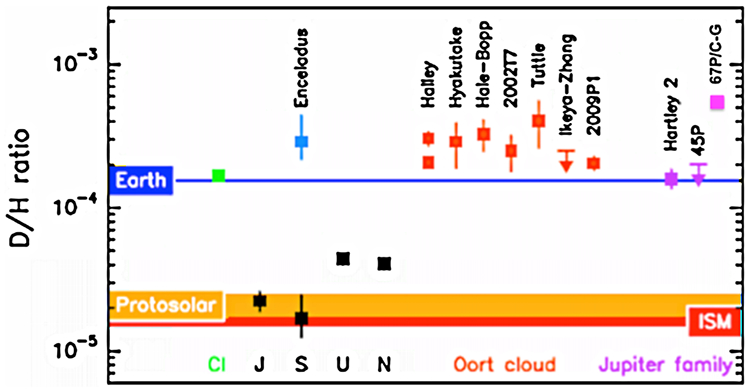
Summary of measured D/H values in the solar system. *Orange symbols*: Oort cloud comets, *purple symbols*: Jupiter family comets. *Black symbols*: measurements in molecular hydrogen in Giant planets. *Blue* and *green symbols* are values in the Enceladus plume and in CI carbonaceous chondrites. ISM and protosolar values are in H_2_ and Earth value is in water. Error bars are 1 sigma. Adapted with permission from Lis et al. (2013). Copyright © 2013 American Astronomical Society

The determination of the D/H ratio in JFCs is a greater challenge because they are generally much fainter than OCCs. The first constraint of D/H in water of a JFC was provided by observations of the Heterodyne Instrument for the Far Infrared (HIFI) (De Graauw et al. 2010) on Herschel (Pilbratt et al. 2010). HIFI is characterized by very high spectral resolution (> 10^6^), near quantum limit sensitivity of its superconductor insulator superconductor (SIS) mixers and the largest collecting area ever flown, i.e. the most sensitive sensor in space for the detection of the D/H ratio thus far. On 17 November 2010, about three weeks after its perihelion, a D/H ratio of 1.61 ± 0.24 × 10^−4^ was derived for the JFC 103P/Hartley 2 from HIFI observations (Hartogh et al. 2011a). This value is compatible with VSMOW and more than 3 times lower than predicted by Kavelaars et al. (2011). Lis et al. (2013) then determined an upper limit for the D/H ratio of the JFC 45P/Honda-Mrkos-Pajdusakova (with HIFI) of 2.00 × 10^−4^, compatible with the result from 103P. Herschel also determined the D/H ratio in the Oort cloud comet C/2009 P1 (Garradd) to be 2.06 ± 0.22 × 10^−4^ (Bockelée-Morvan et al. 2012). Finally, early Rosetta measurements made by the Rosetta Orbiter Spectrometer for Ion and Neutral Analysis (ROSINA) Double-Focusing Mass Spectrometer (DFMS; Balsiger et al. 2007) provide the highest D/H yet measured in a comet, 5.3. ± 0.7 × 10^−4^ (Altwegg et al. 2015). Figure 4 summarizes measurements of the D/H ratio in water and hydrogen throughout the solar system.

The initial finding that the D/H ratio measured in two JFCs did not agree with model predictions followed by the much higher ratio measured by ROSINA in a JFC challenge the existing D/H models, and raise questions as to whether the “classical picture” on the formation regions of OCCs and JFCs is correct. Levison et al. (2010) suggest based on numerical simulations that only 10 % of the OCCs originate in the solar system and perhaps more than 90 % are from protoplanetary disks of other stars. Based on this model, the D/H determined in all OCCs would be expected to show greater scatter from the average value of ~3.00 × 10^−4^. Brasser and Morbidelli (2013) suggest, however, that JFCs and OCCs originated from the same extended outer region of the PSN. This would mean that comets like Hartley 2, which has a lower D/H, formed closer to the Sun and comets with a higher D/H, like 67P/Churyumov-Gerasimenko (hereafter 67P/CG) formed farther out (Altwegg et al. 2015). This is consistent with the scenario suggesting that the migration of the young Jupiter and Saturn towards the inner solar system and back, caused mixing of material over large heliocentric distances (Walsh et al. 2011) causing the original D/H gradient with heliocentric distance to completely vanish and allow similar D/H ratios in both comet families.

Hogerheijde et al. (2011) point in a similar direction, concluding based on considerations on the ortho-to-para ratio (OPR) of water in planet forming disks that comets should contain heterogeneous ice mixtures collected across the entire solar nebula during the early stages of planetary birth. OPR measurements can, within certain limits, be used to determine the spin temperature of water within a cometary coma (Bonev et al. 2007). These spin temperatures are suggested to have been preserved from the time of comet formation (e.g. Crovisier 1984; Mumma et al. 1987), but little is known about the conditions that permit nuclear spin conversion and change the spin temperature of water in comets. Although this conclusion is based on the OPRs determined only in OCCs (e.g. Bonev et al. 2007), measurements for three JFCs (Crovisier et al. 1999; Bonev et al. 2008; Paganini et al. 2012) are consistent with what is observed in OCCs.

More recent D/H models provide alternative scenarios. Albertsson et al. (2014) present results of a laminar model and a model with two-dimensional turbulent mixing taking into account gas-grain chemistry including multiply deuterated species and nuclear spin-states. They find an overlap in the possible formation location for OCCs and JFCs. The model of Yang et al. (2013) does not result in a monotonic increase of the D/H ratio with distance from the Sun, but shows a decrease in the outermost region of the disk. They assume that water equilibrates in the inner disk already during the disk formation phase, when infall of material has not yet stopped. Due to conservation of angular momentum, water that was incorporated early into the disk near the young star would have been pushed outward, carrying low D/H ratios. Since crystalline silicates also formed under high temperature conditions in the inner disk, they predict a correlation between low D/H and a high crystalline/amorphous silicates ratio of comets.

#### 2.3.3 Carbon Isotopes

The ^12^C/^13^C ratio is the best studied of all cometary isotopic ratios with measurements from more than 25 comets that include observations of C_2_, CN, and HCN. Of these, the CN and HCN measurements are the most extensive (e.g. Manfroid et al. 2009), while C_2_ measurements have the longest history (e.g. Danks et al. 1974; Vanysek 1973; Owen 1973; Lambert and Danks 1983), extending from comet Ikeya (1963a) (Stawikowski and Greenstein 1964) through comet C/2002 T7 (LINEAR) (Rousselot et al. 2012). The most recent compilations of data (Jehin et al. 2009; Manfroid et al. 2009 and references therein) include measurements of 23 comets including 8 JFCs and 15 OCCs at heliocentric distances from 1–3 AU and covering multiple compositional classes (e.g. A’Hearn et al. 1995; Fink 2009).

The third carbon species studied is HCN (e.g. Jewitt et al. 1997), which is observed in a different spectral range with different systematic issues than visible CN and C_2_. HCN has been observed at radio wavelengths from the James Clerk Maxwell Telescope (JCMT, Jewitt et al. 1997) and the 12-meter National Radio Astronomy Observatory (NRAO) telescope on Kitt Peak (Ziurys et al. 1999). HCN is the likely parent of CN at large heliocentric distances (Rauer et al. 2003), which introduces a formation bias into the relative isotopic ratios. However, at smaller distances there is at least one additional parent that contributes over a different range of cometocentric distances (e.g. Woodney et al. 2002). Several potential candidates have been suggested ranging from more complex molecular species to direct liberation from dust (e.g. Klavetter and A’Hearn 1994; Woodney et al. 2002; A’Hearn et al. 1986), but to date no definitive source has been identified.

**Fig. 5:**
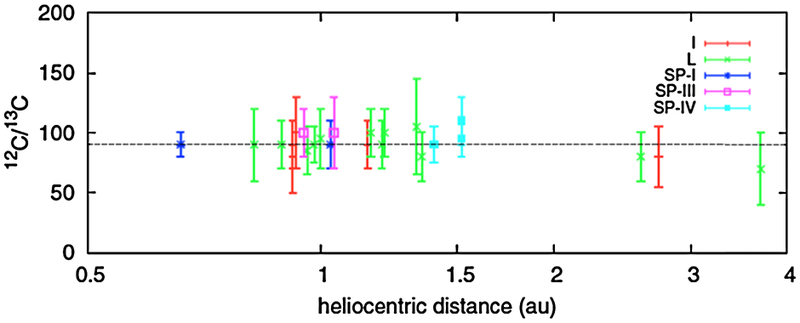
The measured isotopic ratios for carbon are plotted as a function of heliocentric distance and orbital classification—Intermediate-period (I), Long-period (L) and Short-period Tisserand I (SP-I), III (SP-III) and IV (SP-IV). The relative uncertainties overlap for the entire set to a value between 90 and 110, which is consistent with the both the terrestrial value of 89 and the protosolar value of 99.8 (Hashizume et al. 2004). Reprinted from Manfroid et al. (2009) with permission from Astronomy and Astrophysics. Copyright © 2009 ESO

Despite significant systematic and theoretical difficulties associated with the measurements, a remarkable consistency has been obtained in the measurements. Figure 5 summarizes the merged dataset of Manfroid et al. (2009), which includes all of the CN measurements. While there are hints of a potential trend toward a reduction in the ratio with increasing heliocentric distance, within the relative uncertainties of the various measurements obtained from a wide variety of objects with different facilities over several decades is a consistent value for ^12^C/^13^C between 90 and 110 with uncertainties between 10 and 20 %. The C_2_ measurements are similar. All of the measurements are within their relative precision of each other. The majority of objects are slightly above the terrestrial value of 89 and in good agreement with the protosolar value of 99.8 determined from solar wind measurements (Hashizume et al. 2004). However the differences are not statistically significant.

#### 2.3.4 Nitrogen Isotopes

Of the volatile elements other than hydrogen, only nitrogen has an isotopic ratio that is significantly lower in comets compared to the protosolar ^14^N/^15^N value of ~445 (Marty et al. 2011) and also lower than the terrestrial value of 272 (Anders and Grevesse 1989). It has been observed with approximately the same frequency as the carbon ratio since it is present in both CN and HCN, which are the primary species used for carbon studies. The early measurements of the ^14^N/^15^N ratio were obtained from comet C/1995 O1 (Hale-Bopp) using the JCMT and NRAO telescopes (Jewitt et al. 1997; Ziurys et al. 1999). These measurements, 323 ± 46 and 330 ± 98, were both reported as being close to the terrestrial value. However, successive study of CN B-X (0,0) band in the NUV consistently yielded a value for ^14^N/^15^N that was half (i.e. heavily *enriched* in ^15^N) that obtained for HCN (e.g. Arpigny et al. 2003; Manfroid et al. 2009; Hutsemékers et al. 2005; Bockelée-Morvan et al. 2008). This differential between radio and visible measurements extended to Hale-Bopp (Arpigny et al. 2003), which had already been shown to have a consistent isotopic ratio for carbon, as discussed above. Arpigny et al. (2003) suggested that this difference was due to the known changing parentage of CN with heliocentric distance with the contributing parents at small distances having potentially been enriched in ^15^N via an NH_3_ ice-dust grain fractionation process in dense molecular clouds (Charnley and Rodgers 2002).

**Fig. 6:**
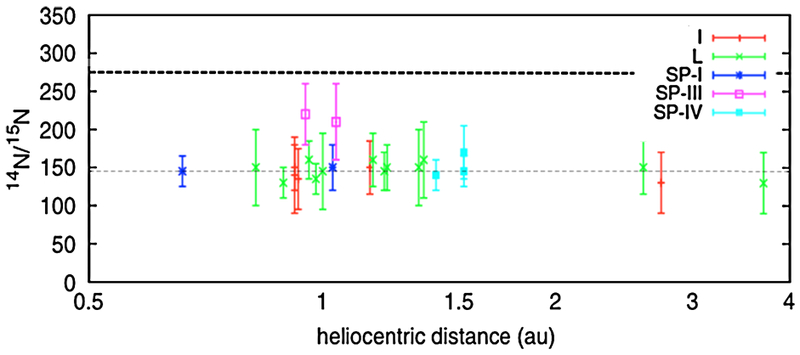
The ^14^N/^15^N ratios of the comets listed in Manfroid et al. (2009) reveals a consistent value for the CN derived number as a function of comet class—Intermediate-period (I), Long-period (L) and Short-period Tisserand I (SP-I), III (SP-III) and IV (SP-IV)—and heliocentric distance. The two HCN values from Hale-Bopp (*pink*) are the least enriched of the entire set. The *thick dashed line* above ^14^N/^15^N = 250 represents the terrestrial value of 272, which does overlap with HCN at the level of their uncertainties. Reprinted from Manfroid et al. (2009) with permission from Astronomy and Astrophysics. Copyright © 2009 ESO

The discrepancy in ^14^N/^15^N may have been resolved by observations of the outburst of 17P/Holmes that were obtained using both the NUV (CN) and radio JCMT (HCN). These observations produced similar results for both species with ^14^N/^15^N in HCN of 139 ± 26, which was only slightly *enriched* relative to ^14^N/^15^N in CN of 165 ± 40 (Bockelée-Morvan et al. 2008). It is important to note that Hale-Bopp and 17/P Holmes are objects in completely different classes (OCC vs. JFC), and the outburst nature of the Holmes event may not have followed the heliocentric distance-parentage relationship observed in other comets. Either factor may have affected the extent of any difference in the ^14^N/^15^N ratio. As a check, Bockelée-Morvan et al. (2008) performed an updated analysis of the original JCMT and NRAO Hale-Bopp data. They identified several contaminating factors that could have biased the original reduction and, after correction, obtained new ratios of 205 ± 70 and 207 ± 48, respectively. While still less enriched than the ratios obtained for CN, the new values were closer to agreement, and to within their relative uncertainties, approximately equal (Fig. 6).

As Fig. 6 shows, the new values are as close to the terrestrial value of 272 as they are to the average of the other measurements. Hence, the possibility of an admixture of CN sources with different ^15^N enrichment factors (Arpigny et al. 2003) cannot be ruled out. This suggests that considerable additional work is required to resolve differences between the terrestrial ^14^N/^15^N ratio and that derived from comets.

Two recent measurements of ^14^N/^15^N in cometary NH_3_ add to the growing dataset: 127 ± 32 (Rousselot et al. 2014) using a summed spectrum of 12 comets, and 139 ± 38 (Shinnaka et al. 2014) from the outburst of comet C/2012 S1 (ISON). These values are in good agreement with the HCN trend of heavy cometary values for ^14^N/^15^N.

#### 2.3.5 Oxygen Isotopes

**Fig. 7:**
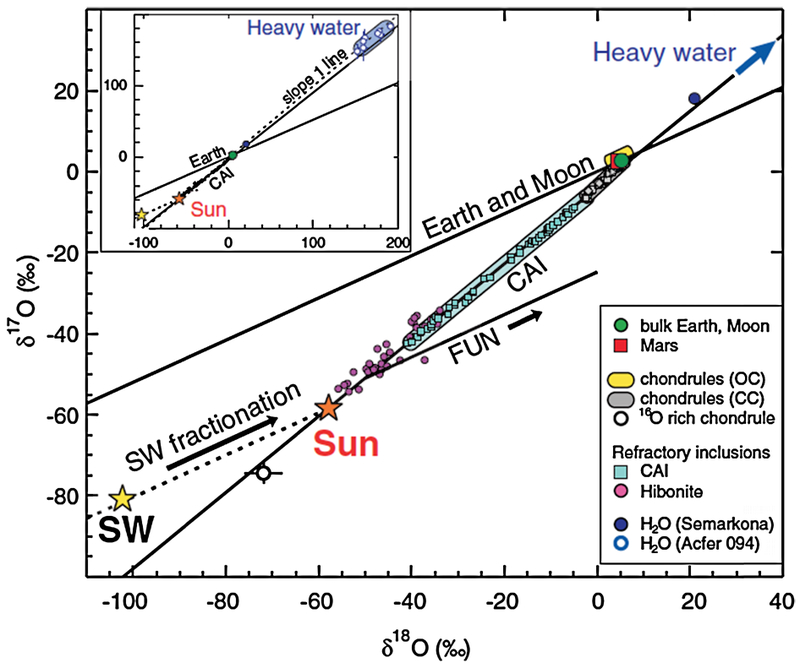
A triple-isotope plot shows relative excesses and deficits relative to SMOW for a variety of astrophysical compounds. The differences come from multiple sources, including, for example, temperature, grain surface chemistry, shock effects, and molecular cloud density. Reprinted with permission from McKeegan et al. (2011). Copyright © 2011 American Association for the Advancement of Science

Oxygen has one of the most diverse set of isotopic measurements of the elements described here, but is also the most complex in terms of its interpretation. It is a major component of terrestrial rocks and interstellar grains with three stable isotopes that occur in ratios that are a strong function of the conditions of their formation (e.g. Webster and Mahaffy 2012; Dominguez 2010). Taken in isolation, the ^16^O/^18^O ratio can vary even if the number for the full system does not. The well-studied variation *δ*^18^O of VSMOW as a function of global terrestrial glaciation, rainfall, and evaporation is a good example of this (e.g. Clark and Fritz 1997). The interaction between different stable isotopes in the formation of different types of oxygen bearing compounds produces a general trend that can be broken down by *δ*^18^O and *δ*^17^O values (Fig. 7) reflecting different processes in the formation and evolution of both the early and later solar system (Dominguez 2010).

The result of these processes presents a situation where comparison with the terrestrial VSMOW value does not provide clear answers on formation and evolution of the solar system. The observed features will reflect the process that formed them (e.g. water ice and CHON grains may have different ^16^O/^18^O values even when obtained from the same body), with Earth itself representing only an emergent property derived from multiple mineralogical and chemical processes acting simultaneously. Because of this, the cometary values should be viewed in the context of whether they fit within the relational framework described in Fig. 7 as opposed to being an exact match to the terrestrial VSMOW.

Cometary ^16^O/^18^O ratios from water of 518 ± 45 and 470 ± 70 at Halley (Balsiger et al. 1995; Eberhardt et al. 1995) and 556 ± 70 at 67P/CG (Altwegg et al. 2015) have been obtained by mass spectroscopy and 530 ± 50 by radio band spectroscopic remote sensing (Lecacheux et al. 2003; Biver et al. 2007). Many other observations of ^16^O and ^18^O in water have also been made with Herschel observations, but are only in few cases converted to a ^16^O/^18^O ratio (e.g. Bockelée-Morvan et al. 2012) due to modeling challenges. Additional water-group spectroscopic measurements have been made of the OH daughter, 425 ± 55 (Hutsemékers et al. 2008). Non-water-group measurements have been made from direct sampling of the mineral grains returned from 81P/Wild2 by *Stardust*, 490–520 (McKeegan et al. 2006). Put within the context of Fig. 7, it is clear that the uncertainties of the measurements defy precision classification with respect to the VSMOW value of 499 in addition to lacking the *δ*^17^O slope indicator. Although the general trend for cometary values is consistent with the range described between chondritic material and asteroidal water, more effective classification will require not only more measurements, but also more precise ones that include ^17^O. Therefore, the oxygen isotope ratios that are currently available are not helpful in evaluating formation and evolution of solar system bodies.

#### 2.3.6 Sulfur Isotopes

**Fig. 8:**
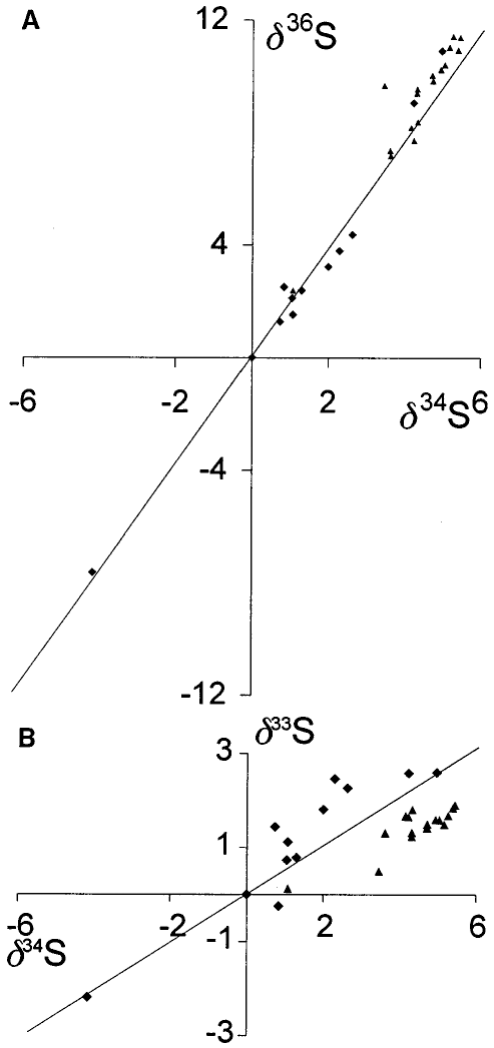
Three isotope plots of *δ*^33^S, *δ*^34^S, and *δ*^36^S are shown for terrestrial samples older than 3 Gyr. The reported cometary values here are generally consistent with the zero point on the *δ*^34^S axis and the lone sample at a significant negative value. Reprinted from Farquhar et al. (2000) with permission. Copyright © 2000 American Association for the Advancement of Science

Like oxygen, sulfur has multiple stable isotopes and its isotopic state is very process dependent. Its mineralogical signature is very different however, with the bulk of its activity being chemical. On Earth, the modern sulfur cycle represents a mixture of biological processes, weathering, and anthropogenic sources (e.g. Farquhar et al. 2000). The best direct comparison with cometary sampling will come from older samples that follow *δ*^33^S, *δ*^34^S, and *δ*^36^S from the modern mean as shown in Fig. 8. Even when focusing on the oldest features, the comparison with cometary values is not straightforward, and given the paucity of measurements, unclear in its significance.

Sulfur isotopes have been sampled in multiple species including plasma (S^+^ mass spectroscopy, Altwegg 1996) and radio-band remote sensing of CS (Jewitt et al. 1997) and H_2_S (Crovisier et al. 2004). All are focused on the ^32^S/^34^S and its comparison with the modern value of ~23. The resulting values are nominally consistent with the terrestrial value, but do have certain inconsistencies. In particular, the ^32^S/^34^S ratios in CS and H_2_S from Hale-Bopp of 27 ± 3 and 16 ± 3, respectively, are highly disparate. However, since they come from different species they may be process specific.

The ^32^S/^34^S ratio in CS has also been observed to vary, with the value from Hale-Bopp of 27 ± 3 being very different from that obtained during the outburst of 17P/Holmes of 16 ± 3. Aside from the previously noted difference in both the evolutionary state and population (dynamically new OCC vs. JFC) and activity cycle (heliocentric driven vs. outburst) it is difficult to make any quantitative conclusions about this difference, particularly given the small sample size of sulfur measurements. The similarity between the H_2_S value from Hale-Bopp and the CS value from Holmes may be indicative of an underlying process, but until a larger sample size is obtained that targets multiple isotopes from several comets of different classes and heliocentric distances, any conclusions outside of a general agreement with terrestrial averages is premature. Therefore, as with the oxygen isotope ratios the sulfur isotope ratios that are currently available are not helpful in evaluating formation and evolution of solar system bodies.

#### 2.3.7 Noble Gases

Noble gases and their isotopologues are inert and not subject to chemical processes. Their abundance in comets, meteorites and atmospheres can serve as a valuable tracer of conditions within the PSN, processes that deliver volatiles to solar system bodies and processes that change atmospheres with time. Unfortunately at the present time the only available measurements of noble gases in comets are upper limits and a tentative detection of Argon (Stern et al. 2000). Noble gas measurements in chondrites and terrestrial planet atmospheres compared to solar abundances have been used to evaluate the origin of volatiles on the terrestrial planets (e.g. Owen and Barnun 1995) and the evolution of the terrestrial planet atmospheres (e.g. Pepin 1991).

**Fig. 9:**
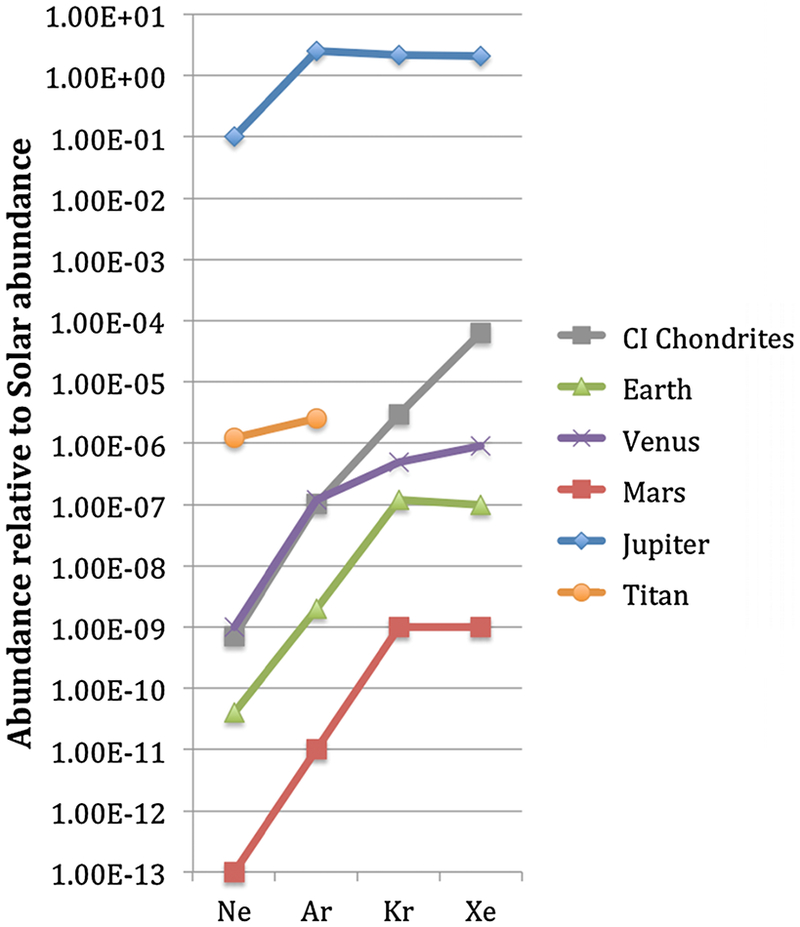
Noble gas abundances relative to solar noble gas abundances for Jupiter, CI Chondrites, Titan and the terrestrial planets

Figure 9 illustrates the noble gas measurements available for planets, for CI chondrites and for one of the moons in the solar system relative to the solar abundance of noble gases. Titan’s abundance is determined based on noble gas abundance relative to nitrogen compared to solar abundance relative to nitrogen assuming that the ratio of ^22^Ne to ^20^Ne is solar. The remaining ratios are determined based on the abundance of noble gases to 10^6^Si atoms.

Jupiter’s noble gas abundances are greater than the solar abundances while the terrestrial planets, CI chondrites and Titan are significantly depleted in noble gases. All of these bodies are most depleted in Ne compared to solar values and a trend of increased depletion with decreasing mass is observed in Titan, the terrestrial planets and CI chondrites. These depletion patterns could provide information about the potential sources of volatiles for these bodies, although the present lack of measurements in comets is very limiting.

## 3 The Giant Planets

Here we review the atmospheric elemental and isotopic compositions of Jupiter, Saturn, Uranus and Neptune. We then discuss the constraints that can be derived on the formation conditions of these giant planets in the protosolar nebula from their inferred compositions.

### 3.1 Atmospheric Composition

The abundances and isotopic ratios of most significant volatiles measured at Jupiter, Saturn, Uranus and Neptune are summarized in Table 3. Elemental abundances have been expressed as a function of the protosolar abundances derived from Lodders et al. (2009). The abundances of CH_4_, NH_3_, H_2_O, H_2_S, Ne, Ar, Kr and Xe were measured by the Galileo Probe Mass Spectrometer (GPMS) in Jupiter’s atmosphere (Mahaffy et al. 2000; Wong et al. 2004). The value of H_2_O abundance reported for Jupiter corresponds to the deepest measurement made by the probe (at ~20 bar). It is probably smaller than the planet’s bulk water abundance, which remains unknown (Atreya et al. 2003; Wong et al. 2004; Mousis et al. 2012). The He abundance in Jupiter has also been measured in situ by a Jamin-Mascart interferometer (Helium Abundance Detector) aboard the Galileo probe (von Zahn and Hunten 1992; von Zahn et al. 1998). PH_3_ is the only species of our list of measurements in Jupiter whose abundance has been determined remotely by the Cassini Composite Infrared Spectrometer (CIRS) during the spacecraft encounter in 2000–2001 (Fletcher et al. 2009a). The GPMS instrument aboard the Galileo probe has also performed the isotopic measurements presented for Jupiter in Table 3 (Niemann et al. 1996, 1998; Mahaffy et al. 2000; Wong et al. 2004).

In the case of Saturn, only the abundances of CH_4_, PH_3_, NH_3_, H_2_O, He, and indirectly that of H_2_S, have been determined. The abundance of CH_4_ has been measured from the analysis of high spectral resolution Cassini/CIRS data (Fletcher et al. 2009b). Similar to Jupiter, PH_3_ has been determined remotely in Saturn from Cassini/CIRS observations (Fletcher et al. 2009a). The NH_3_ abundance corresponds to the deepest value derived by Fletcher et al. (2011a) who examined Saturn’s tropospheric composition from Cassini/VIMS thermal emission spectroscopy. Tropospheric H_2_O has been inferred in Saturn via the Short Wavelength Spectrometer Instrument onboard the Infrared Space Observatory (ISO) (de Graauw et al. 1997). However, H_2_O is unsaturated at this altitude (~3 bar level), implying that its bulk abundance should be higher than the measured one. The H_2_S abundance is quoted from the indirect determination of Briggs and Sackett (1989) who investigated the influence of models of NH_3_-H_2_S-H_2_O cloud decks on Saturn’s atmospheric opacity at microwave wavelengths. The He abundance in Saturn’s atmosphere comes from a reanalysis of Voyager’s infrared spectrometer (IRIS) measurements (Conrath and Gautier 2000). The only isotopic ratios measured in Saturn are D/H in H_2_ (Lellouch et al. 2001) and ^12^C/^13^C in CH_4_ (Fletcher et al. 2009b).

Due to their higher heliocentric distances, the measurements of molecular abundances and isotopic ratios are scarce in Uranus and Neptune. CH_4_ has been measured in the atmospheres of the two planets with the Hubble Space Telescope (HST) Space Telescope Imaging Spectrograph (STIS) (Karkoschka and Tomasko 2009, 2011). The H_2_S abundance has been indirectly inferred in Uranus and Neptune from the fits of thermal spectra with models including the microwave absorption of this molecule (de Pater et al. 1991). N has not been measured in the atmospheres of the two ice giants, but its equilibrium form should be NH_3_ in the troposphere, as is the case for Jupiter and Saturn (Fegley and Prinn 1986). The He abundance in the two planets has been inferred from the combination of Voyager radio-occultation and infrared spectroscopy measurements (Conrath et al. 1987, 1991).

### 3.2 Interpretations of the Volatile Enrichments in the Atmospheres of Jupiter, Saturn, Uranus and Neptune

Table 3 shows that C, N, P, S, Ar, Kr and Xe are found to be uniform and enriched by a factor from ~2 to 4 in Jupiter. In contrast, C, N and P (the only heavy elements reliably measured) are found to be enriched by factors of ~10, 3 and 12 respectively in Saturn. Helium is depleted compared to the protosolar value in Jupiter and Saturn because of its condensation into droplets that precipitate in the deep interiors of the two giants (Fortney and Hubbard 2003). Interestingly, the Galileo probe entry site in Jupiter’s atmosphere was an unusually dry meteorological system. This implies that the probe did not measure the deep, well-mixed water abundance (Wong et al. 2004).

An interpretation of the volatile enrichments measured in the four giant planets is that their building blocks agglomerated from a mixture of rocks and crystalline ices that condensed during the cooling period of the protosolar nebula (Gautier et al. 2001; Hersant et al. 2004, 2008; Alibert et al. 2005a, 2005b; Gautier and Hersant 2005; Mousis et al. 2006, 2009c, 2010, 2012; Marboeuf et al. 2008, 2014a, 2014b). The ices consist of a mixture of clathrates or hydrates (case of NH_3_) and pure condensates whose relative proportions were fixed by the availability of crystalline water at the time of volatiles trapping in the clathrate phase and the efficiency of the clathration kinetics in the protosolar nebula (Mousis et al. 2009c). In order to explain the uniform enrichment of volatiles in Jupiter’s atmosphere, it has been proposed that the abundance of water ice was approximately twice as large as the protosolar O abundance (Gautier et al. 2001). In these conditions, essentially all volatiles, including noble gases, were trapped by water in the ~45–80 K range in the form of clathrates or hydrates instead of condensing as pure ices at lower temperatures in the protosolar nebula (Gautier et al. 2001). The volatile enrichments in Jupiter can then be explained by the accretion and the vaporization in its envelope of the icy planetesimals formed in its feeding zone. Alternative models suggest that the building blocks of Jupiter may have formed in a cooler disk (down to ~20 K), implying the relaxing of the condition of a supersolar O abundance that is needed to form clathrates at higher temperatures (Mousis et al. 2009c, 2012). In this case, the assumption of a protosolar O abundance in the nebula would imply that the fraction of pure condensates would be higher than that of clathrates in planetesimals.

However, more recent work suggests that both Jupiter and Saturn were formed in environments where the O abundance was supersolar. Indeed, the scenario of full clathration of volatiles explains their observed enrichments in the two giants and is also found consistent with the high ^14^N/^15^N ratios measured in their atmospheres (Mousis et al. 2014a). Consequently, these values require significant amounts of N_2_ to have been trapped in the building blocks of the two planets at higher temperatures in the disk than those required for the condensation of this volatile (~22 K). The scenario of clathration of volatiles also matches the observational data of Uranus and Neptune (Gautier and Hersant 2005) but the known constraints on these two planets are too scarce and can also be explained by alternative scenarios. For example, it has been proposed that Uranus and Neptune could have formed between the CO and N_2_ ice lines, assuming that the disk was stationary (Ali-Dib et al. 2014). This model explains the D/H value found in both planets, matches well the heavy C enrichment in the two planets and predicts that N is protosolar in the envelopes. However, it requires a homogenous envelope while many internal structure models suggest a non-homogenous envelope. Clathration models predict that nitrogen is moderately enriched in Uranus and Neptune (Gautier and Hersant 2005).

### 3.3 Contribution of Comets to the Giant Planet Atmospheres

While comets provide us with invaluable information on the Solar System formation conditions from their composition, they are also thought more and more to be active players in the temporal evolution of giant planet atmospheric composition. This section reviews the current knowledge regarding the contribution of comets to the composition of giant planet atmospheres.

#### 3.3.1 An Emblematic Case: The Shoemaker-Levy 9 Comet Impacts of 1994 with Jupiter

The Shoemaker-Levy 9 comet (SL9; Shoemaker et al. 1993), also known as the “String of Pearls” comet because of its fragmented form, collided with Jupiter in July 1994 and left visible scars on the Jovian disk for several weeks. This spectacular event was the first direct observation of an extraterrestrial collision in the Solar System and was followed worldwide by professional and amateur astronomers.

Observations of the impacts over a wide range of wavelengths led to the detection of several species, including H_2_O, CO, OCS, CS, CS_2_, S_2_, HCN, and NH_3_, in Jupiter’s stratosphere at the impact sites (e.g., Bjoraker et al. 1996; Lellouch et al. 1995; Atreya et al. 1995; Noll et al. 1995; Marten et al. 1995; Orton et al. 1995). While the presence of NH_3_ was likely due to the updraft of air parcels from the NH_3_-rich upper troposphere following the impacts (Orton et al. 1995), the formation of most of these species was explained by shock chemistry occurring at extreme temperatures during the impacts (Zahnle 1996). Post-impact high temperatures were still observable a few days after the impacts (Orton et al. 1995; Bézard et al. 1997a, 1997b; Lellouch et al. 1997; Moreno et al. 2001).

Comets are best known for being water-rich bodies (e.g., Bockelée-Morvan 2011). Therefore, the detection of water vapor in the stratospheres of the giant planets (i.e., above the water ice cloud that prevents any transport of water vapor from their water-rich deep atmospheres up to observable levels) by the ISO (Feuchtgruber et al. 1997) was less of a surprise in Jupiter than in the other giant planets: SL9 was indeed a perfect candidate source as its fragments had disintegrated in Jupiter’s stratosphere a few years earlier. However, other possible sources for external water exist in the outer solar system: interplanetary dust particles (Prather et al. 1978), which result from comet activity and asteroid collisions, and icy rings and satellites (Strobel and Yung 1979). Another complication comes from the results of shock chemistry simulations, according to which most of the cometary H_2_O had converted into CO during the impact (Zahnle 1996). So, while H_2_O was directly detected at some impact sites by Bjoraker et al. (1996), subsequent ISO Submillimeter Wave Astronomy Satellite (SWAS), and Odin observations did not lead to a direct proof that Jupiter’s stratospheric H_2_O had been delivered by SL9, because of limitations in spatial resolution, spectral resolution, and sensitivity (Lellouch et al. 2002; Bergin et al. 2000; Cavalié et al. 2008, 2012). Only recently has Herschel been able to demonstrate that the spatial distribution of H_2_O was a remnant of the SL9 impacts of 1994 (Cavalié et al. 2013).

The mid- and long-term consequences of such enrichment in the chemical inventory of Jupiter’s stratosphere were investigated by means of photochemical models (Moses et al. 1995a, 1995b; Moses 1996). According to these simulations, the most stable species over the long-term (i.e., several years) after such impacts are CO, CS, HCN, and to a lesser extent H_2_O. All of these species have been monitored since that time and used as atmospheric tracers to constrain vertical and horizontal transport, and Jovian oxygen photochemistry (Moreno et al. 2003; Griffith et al. 2004; Lellouch et al. 2006; Cavalié et al. 2008).

#### 3.3.2 Beyond Jupiter

The comet impact rates in the Solar System have been studied since long before the SL9 events (e.g., Everhart 1969; Zimbelman 1984; Olsson-Steel 1987). Levison et al. (2000) and Zahnle et al. (2003) provided the latest estimates for the Giant Planets based on cratering rates. Work more focused on Jupiter has been published since that time (Sánchez-Lavega et al. 2010; Hueso et al. 2013).

CO is one of the most stable post-SL9 species in Jupiter’s stratosphere with respect to its abundance (Moreno et al. 2003), and it is still monitored as of 2014. It is therefore no surprise that tentative evidence for comet impact signatures has been found in giant planets beyond Jupiter using observations of this species. The observability of cometary-derived species like CO is made plausible given the existing trade-space found between km-size comet impact rates at Jupiter, Saturn, Uranus, and Neptune (~0.01–0.001 year^−1^; Zahnle et al. 2003), and stratospheric meridional/vertical mixing timescales (~100–1000 years; Moreno et al. 2003; Moses et al. 2005). This trade-space seems to ensure that comets of decent size can leave observable signatures on the long term in giant planet stratospheres.

In the past decade, observations of CO in the millimeter and submillimeter ranges at Saturn and Neptune have tentatively shown that the external source for this species may be ancient comet impacts (Lellouch et al. 2005, 2010a; Hesman et al. 2007; and Luszcz-Cook and de Pater 2013, for Neptune, and Cavalié et al. 2010 for Saturn). At Uranus, a cometary origin for CO is possible (Cavalié et al. 2014). These results are based on spectrally resolved ground- and space-based observations and vertical transport modeling.

To account for Neptune’s observed external CO, the required CO flux is 10–500 times that of H_2_O, i.e., the same order of magnitude as in Jupiter, thus favoring comet impact shock chemistry as the formation process for Neptune’s external CO (Lellouch et al. 2005). In addition, HCN is also present in Neptune’s stratosphere (Marten et al. 1993) and the CO/HCN ratio is similar to post-SL9 values. Lellouch et al. (2005) concluded that HCN and CO might therefore derive from the same comet.

At Saturn, there is as yet no additional clue (beyond CO) regarding the presence of cometary-derived material. HCN remains undetected and other oxygen species probably originate from the Enceladus geysers and subsequent transport in the Saturnian system (Waite et al. 2006; Cassidy and Johnson 2010; Hartogh et al. 2011b). At Uranus, the origin of other oxygen species is still under investigation (Orton et al. 2014).

In the cases of Saturn and Neptune, ~km-size comets impacting the planets a few centuries ago are required to fit the CO data. One should note that Bézard et al. (2002) had already demonstrated that Jupiter’s CO vertical profile showed evidence for pre-SL9 comet impacts.

#### 3.3.3 New Impacts?

In the last few years, amateur observations have led to the unexpected detection of new impacts in Jupiter’s atmosphere in 2009, 2010, and 2012 (Sánchez-Lavega et al. 2010; Fletcher et al. 2010, 2011b; Hueso et al. 2010a, 2013). While the size of the 2009 impactor (~0.5 km) made the impact look like one of the main SL9 impacts, the high abundance of silica and the lack of spectroscopic evidence of cometary-derived species like CO, CS, and HCN, favor an asteroidal origin rather than a cometary origin for this impactor (Orton et al. 2011).

In the future, nearly continuous monitoring of Jupiter offered by the extensive amateur network (Hueso et al. 2010b; Mousis et al. 2014b), will undoubtedly lead to more impact detections and to a better understanding of the contribution of comets to the composition of giant planet atmospheres.

The study of the contributions of comets to the composition of giant planet atmospheres will not only benefit the Solar System atmosphere field but also the growing field of exoplanet atmosphere characterization (Turrini et al. 2014).

## 4 Titan, Enceladus and Giant Planet Satellites

The giant planet satellites are divided into two categories depending on their formation process: (1) regular satellites formed as a by-product of giant planet formation and (2) irregular satellites formed in the PSN that were captured by their planet. Captured satellites of giant planets are likely to have formed in conditions similar to objects in the Kuiper Belt and Oort Cloud. Their composition would provide important information about their source region and dynamical processes in the early solar system.

Understanding of the formation conditions of the regular satellites has progressed along with studies of giant planet formation. Initially, giant planets were thought to have formed within a subnebula that was significantly warmer than the PSN and dense enough to convert CO and N_2_ from the PSN to CH_4_ and NH_3_ (Prinn and Fegley 1989). However, Canup and Ward (2002) found that the subnebula of Jupiter must have had a gas density that was orders of magnitudes lower than previously thought. Therefore, the subnebula was probably not dense enough to efficiently convert CO to CH_4_ and N_2_ to NH_3_ (Mousis et al. 2002; Alibert and Mousis 2007). Canup and Ward (2006) then showed that the regular satellites formed within a circumplanetary disk that was actively supplied with gas and solids from the PSN, and that the earliest satellites to form were lost by collision with the giant planets. The regular satellites that remain today are proposed to have formed during the latest stages of planetary formation in a “gas-starved” disk (Canup and Ward 2006).

The composition of regular satellites is an important tool for constraining the effective temperature of the subnebula and thus the inflow properties during the formation of the giant planet (Canup and Ward 2006). Satellites that are formed early in the process of giant planet formation are predicted have a low ice-to-rock ratio compared to those that formed later, so the high ice-to-rock ratio of most of the giant planet satellites is interpreted to mean that they formed later in the process of planetary formation (Canup and Ward 2006). However, it is also possible that these satellites formed at a greater distance from their parent planet and migrated to their current position (Mousis et al. 2009a; Crida and Charnoz 2012). Comparing the primordial composition and isotopic ratios of noble gases and carbon, nitrogen, hydrogen and oxygen to solar composition and the composition of comets provides important clues as to the formation conditions of these satellites.

### 4.1 Enceladus

Enceladus is a small moon (radius of ~500 km) located within the E ring of Saturn. A thick layer of high albedo water ice covers the surface. The Cassini mission discovered a water-rich plume emanating from the south pole (Porco et al. 2006; Hansen et al. 2006; Spencer et al. 2006), the composition of which has been evaluated by the Cassini Ion Neutral Mass Spectrometer (INMS) (Waite et al. 2006; 2009). This composition includes volatiles that could either be primordial or produced by aqueous processes (Matson et al. 2007; Glein et al. 2008). Uncertainty in the source of some molecules found in the plume of Enceladus limits the use of its composition for direct comparison with formation processes. For example, the origin of CH_4_ detected in the plume could be primordial or the methane may have been produced through serpentinization reactions in the interior.

One measurement from the plume that can be presumed to be primordial is the D/H ratio in H_2_O. This was measured to be $2.9_{-0.7}^{+1.5}$ × 10^−4^ (Waite et al. 2009), which is very close to the value found for OCCs (see Fig. 4), and serves as the most useful tool currently available for evaluating the formation of Enceladus.

When evaluating the formation of Saturn’s moon Titan, Alibert and Mousis (2007) proposed that the D/H in water for Saturn’s moons would show a gradient based on their formation region. Moons that formed closer to Saturn would have a lower D/H than moons that formed farther away or those that were captured. However, a D/H ratio for Enceladus that is in the range of OCCs strongly indicates that the building blocks for Enceladus must have formed in the PSN (Mousis et al. 2009b). These building blocks are proposed to have migrated inward through the Saturnian subnebula where they were partially devolatilized, losing primordial Ar, CO and N_2_ in the process (Mousis et al. 2009b) in agreement with the model of Canup and Ward (2002, 2006).

### 4.2 Titan

Table 4 lists measurements made in Titan’s atmosphere that are relevant to studying Titan’s formation and evolution compared to measurements made in comets and estimates for PSN values. While each of these measurements provides insight into the evolution of Titan’s atmosphere, the most important values for evaluating Titan’s formation are the three that have been determined to represent primordial composition of the building blocks of Titan (italicized in Table 4). The D/H in water at Titan can be presumed to be in the same range as was determined for Enceladus (Waite et al. 2009; Mousis et al. 2009a). The limited range of values for primordial D/H in methane is based on limits placed by the evolution of ^12^C/^13^C in Titan’s methane (Mandt et al. 2009, 2012), while the upper limit for ^14^N/^15^N in Titan’s nitrogen inventory is based on the maximum possible fractionation allowed due to escape processes (Mandt et al. 2014).

The primordial forms of carbon and nitrogen in the building blocks of Titan provide important clues to the conditions in which Titan formed. The ratio of CO to CH_4_ in Titan’s atmosphere (Gautier and Raulin 1997) is orders of magnitude lower than the ratio determined for the PSN (Lodders 2003), suggesting either that Titan’s building blocks were initially enriched in CH_4_ (Lunine 1989) or that the building blocks were rich in CO that was later converted to CH_4_ through aqueous processes in the interior (Atreya et al. 2006). The nitrogen could have originated as N_2_ or as NH_3_ that was later converted to N_2_ by photochemical processing in the early atmosphere (Atreya et al. 1978), impact shock heating (McKay et al. 1988), or endogenic processes (Glein et al. 2008).

The original thought that Titan formed in a warm subnebula that was dense enough to convert CO and N_2_ from the PSN to CH_4_ and NH_3_ (Prinn and Fegley 1989) would have produced building blocks that preferentially trapped CH_4_ and NH_3_ for two reasons. The first reason is that CH_4_ and NH_3_ would have been more abundant than CO and N_2_ in the Saturnian subnebula (Prinn and Fegley 1989). However, in a gas-starved disc (Canup and Ward 2002, 2006), CO and N_2_ would be more abundant than CH_4_ and NH_3_ because the gas density is too low for conversion to be effective (Mousis et al. 2002; Alibert and Mousis 2007).

The second reason that the building blocks could preferentially trap CH_4_ and NH_3_ in the Saturnian subnebula is that the temperature could have been too high to trap CO and N_2_, which are trapped at much lower temperatures than CH_4_ and NH_3_ (Bar-Nun et al., 1985, 1988). This possibility would still be valid in a gas-starved disc and is further supported by the measured ^36^Ar abundance relative to N_2_ of 1.1 × 10^−7^ (Niemann et al. 2010). Like CO and N_2_, ^36^Ar is trapped at very low temperatures. A measured abundance that is orders of magnitude lower than the solar abundance of ~0.04 suggests that Titan’s building blocks may have formed in temperatures as high as 100 K (Owen 2008).

However, the ^36^Ar/N ratio could also be explained by the removal of ^36^Ar from the atmosphere by processes such as trapping in surface clathrates (e.g. Fortes and Stofan 2005; Osegovic and Max 2005; Mousis et al. 2011). Furthermore, if Titan had formed at such high temperatures, the D/H in water ice of Titan would be lower than that of OCCs (Owen 2008), which disagrees with the D/H measurement at Enceladus (Mousis et al. 2009a). As shown in Table 4, we can assume that Titan and Enceladus formed under similar conditions and that Titan’s D/H in H_2_O is also similar to that of OCCs (Mousis et al. 2009a). A D/H similar to that of OCCs requires very low formation temperatures, like those found in the PSN, and excludes the possibility of formation temperatures above 100 K. This suggests that N_2_ and CO could have initially been trapped in the building blocks of Titan. Further constraints are, therefore, needed to determine the primordial forms of carbon and nitrogen in Titan’s building blocks.

The primordial D/H in methane and ^14^N/^15^N in nitrogen provide important clues as to the origin of carbon and nitrogen on Titan. Based on the evolution of ^12^C/^13^C in methane, the primordial D/H in methane is distinctly lower than the water D/H, and must have been between 9.5 × 10^−5^ and 1.6 × 10^−4^ (Mandt et al. 2009, 2012; Nixon et al. 2012). If the methane in Titan’s atmosphere had been formed by serpentinization reactions in the interior (e.g. Atreya et al. 2006) then the primordial D/H ratio in methane would be much higher due to the high D/H in Titan’s water. A lower primordial D/H suggests, then, that Titan’s methane is primordial (Mousis et al. 2009a). Furthermore, limitations on the evolution of ^14^N/^15^N in Titan’s atmosphere place the primordial ^14^N/^15^N for Titan well within the range for primordial NH_3_ in the PSN (Mandt et al. 2014), suggesting that Titan’s nitrogen originated as NH_3_. These constraints point to an origin of Titan’s building blocks in the PSN (Mousis et al. 2009a; Mandt et al. 2014), but still require depletion in N_2_, CO and possibly ^36^Ar. This depletion can be explained by migration of the building blocks from the PSN into the warmer Saturnian subnebula (Canup and Ward 2002, 2006) where some partial devolatilization occurred (Mousis et al. 2009a; Mandt et al. 2014).

### 4.3 The Galilean Satellites

The four largest satellites of Jupiter—known as the Galilean satellites because of their initial discovery by Galileo in 1610—are Io, Europa, Ganymede and Callisto. Io, located closest to Jupiter, is subjected to significant tidal heating due to its orbital resonance with Jupiter and the other satellites, leading to extensive volcanic activity. The other Galilean satellites show evidence in their surface structures of thermal processing due to tidal heating, which decreases with increasing distance from Jupiter. Both Europa and Ganymede are found to be tectonically active due to tidal heating and have experienced extensive resurfacing. Callisto is subjected to the least amount of tidal heating and has a surface that is covered in impact craters suggesting little to no geological activity over its history (Estrada et al. 2009). The primordial composition of Io is difficult to constrain due to the extensive heating to which it has been subjected, but Europa, Ganymede and Callisto have all been able to retain substantial amounts of water ice throughout their history. It is unclear how much water ice Io may have initially contained.

As discussed earlier, the regular Jovian satellites are presumed to have formed in a circumplanetary nebula that was accreting material from the PSN (Canup and Ward 2002). Models of the temperature and pressure profile initially suggested that water ice was accreted starting at or just beyond the orbit of Europa, the second closest Galilean satellite (e.g. Lunine and Stevenson 1982), and that the ice-to-rock ratios of Jupiter’s satellites indicate their formation distance within the subnebula (e.g. Canup and Ward 2002; Mousis and Gautier 2004). However, the discovery that Amalthea, a small regular moon orbiting closer to Jupiter than Io, had a higher ice-to-rock ratio than the Galilean satellites (Anderson et al. 2005) challenged this assumption. This discovery brought about the possibility that all of the Jovian moons could have accreted significant amounts of ice, and that subsequent heating—either tidal heating or bombardment—could have driven off much of the water ice from Io and Europa (Estrada et al. 2009). However, this does not rule out the possibility that Amalthea formed later than the giant satellites or in a colder region of the subnebula and migrated to its current location (Anderson et al. 2005).

Other than the ice-to-rock ratio, the only available composition measurements for the Galilean satellites is surface composition, from which no isotopic or noble gas information is currently available. Galileo magnetic field measurements have found indications of subsurface oceans on Europa (Khurana et al. 1998; Kivelson et al. 1999), Ganymede (Kivelson et al. 2002) and Callisto (Khurana et al. 1998; Kivelson et al. 1999) where endogenic chemistry is likely to have influenced the composition of these internal oceans. The extensive resurfacing of Europa and Ganymede suggest exchange between an interior ocean and the surface. Therefore, it is difficult with the little information available on surface composition to determine the primordial forms of carbon and nitrogen in order to constrain formation scenarios for these satellites. Furthermore, the surfaces of all four satellites are subjected to extensive radiolitic chemistry caused by energetic particles in Jupiter’s magnetosphere (Johnson et al. 2004) that modifies the surface composition. One thing to note is that the possible presence of liquid water below the surface suggests the possible presence of ammonia (e.g. Kargel 1992). However, it is difficult to know if either of this is primordial because NH_3_ could be present as a result of endogenic chemistry. Therefore, little can be said about the formation processes of the Galilean satellites based on their composition beyond the fact that they must have accumulated significant amounts of water ice during their formation, as was predicted by Canup and Ward (2002, 2006).

### 4.4 Other Satellites of Interest

Data on the composition of the other outer planet satellites is very limited. CO_2_ is frequently seen on the surface of outer planet moons, but many possibilities exist for *in situ* production of this molecule. It has been observed on the surface of Saturn’s satellites Phoebe (Clark et al. 2005), Iapetus (Buratti et al. 2005) and Hyperion (Cruikshank et al. 2007) as well the Uranian moons Ariel, Umbriel and Titania (Grundy et al. 2006). As with Jupiter’s icy moons, it is currently unclear if this CO_2_ is primordial or produced through surface chemistry or endogenic processes so no conclusion can be made about the formation processes of these satellites based on this measurement.

Neptune’s moon Triton has an orbit that is retrograde and highly inclined, suggesting that it is a captured moon presumed to have originated in the Kuiper Belt (Goldreich et al. 1989; Agnor and Hamilton 2006). Triton’s surface is made up primarily (~55 %) of N_2_ ice with water and CO_2_ ices making up the remaining ~45 %. Trace amounts of CH_4_ and CO have also been detected on its surface (McKinnon and Randolph 2014) as well as in the atmosphere (Lellouch et al. 2010b). The source of the N_2_ ices, whether N_2_ or NH_3_ in the PSN, can provide important information about the formation conditions of Triton and, by extension, Kuiper Belt objects. Nitrogen isotope ratio measurements, in particular, would be of high value.

Understanding of the composition of comets has played an important role in determining the origin of the Saturnian satellites based on the D/H measurement in water at Enceladus (Mousis et al. 2009b) and the ^14^N/^15^N in N_2_ and D/H in CH_4_ at Titan (Mousis et al. 2009a; Mandt et al. 2014). Further isotopic measurements in comets and in the outer planet satellites, as well as noble gas measurements in each of these solar system objects will play an important role in constraining the origins of other outer planet satellites as well as the giant planets.

## 5 The Terrestrial Planets

A cometary origin for the terrestrial oceans is often advocated in the literature. It makes sense to assume that terrestrial water originated from impacts of bodies like comets, which are otherwise known to be water-rich and which periodically cross the inner solar system. Modern versions of these scenarios arise from dynamical models advocating large-scale injection of small bodies formed beyond the snow line into the inner solar system following disruption of giant planet trajectories (Walsh et al. 2011). However, only Earth has a significant amount of water at its surface, while the other terrestrial bodies (Mercury, Venus, Moon, Mars) are dry or highly water-depleted. Such depletions could be the result of atmospheric escape processes and not necessarily the result of heterogeneous distribution of water contributors, but even the Earth contains a significantly small amount of water: the oceans are equivalent to about 260 ppm H_2_O normalized to the mass of our planet, and the bulk Earth that includes deep reservoirs may contain no more than about 1000–3000 ppm (Marty 2012), which is still low compared to comets (~50 % water) or the other possible contributors that are wet asteroids.

Carbonaceous chondrites contain up to 15–20 % equivalent water in the form of hydrated minerals, which is not far from cometary concentrations. Thus a small contribution of either wet asteroidal material (e.g., 0.8–2 % carbonaceous chondrite-type; Marty 2012), or cometary matter (less than 0.5 %) could account for the water inventory of Earth. Alternatively the Earth and inner planets could have grown up from wet material from the beginning, but this possibility is at odds with the reduced character of Earth’s building blocks that permitted formation of an iron-rich core. Thus current models advocate a proto-Earth (and inner planets) being built first from reduced, and supposedly dry, material, followed by increasing contributions of more oxidized contributors (e.g. Wänke and Dreibus 1988). This type of scenario is consistent with dynamical models in which growing inner planets become wet with time (e.g. Morbidelli 2002).

### 5.1 Asteroidal Versus Cometary Origin for Inner Planet Volatiles: Abundances

Two types of very volatile elements can be used as diagnostics of the origin(s) of the inner planet atmospheres. Here atmosphere means the inventory of volatile elements at the surface of planets, that is, atmosphere stricto sensu plus the oceans and sediments for Earth. On the one hand, the noble gas elemental and isotopic compositions are key tracers of origin and physical processes due to their chemical inertness. On the other hand, some of the stable isotope ratios of light elements, specifically those of H and N, show dramatic variations among solar system objects and reservoirs that make them unique cosmochemical tracers.

**Fig. 10:**
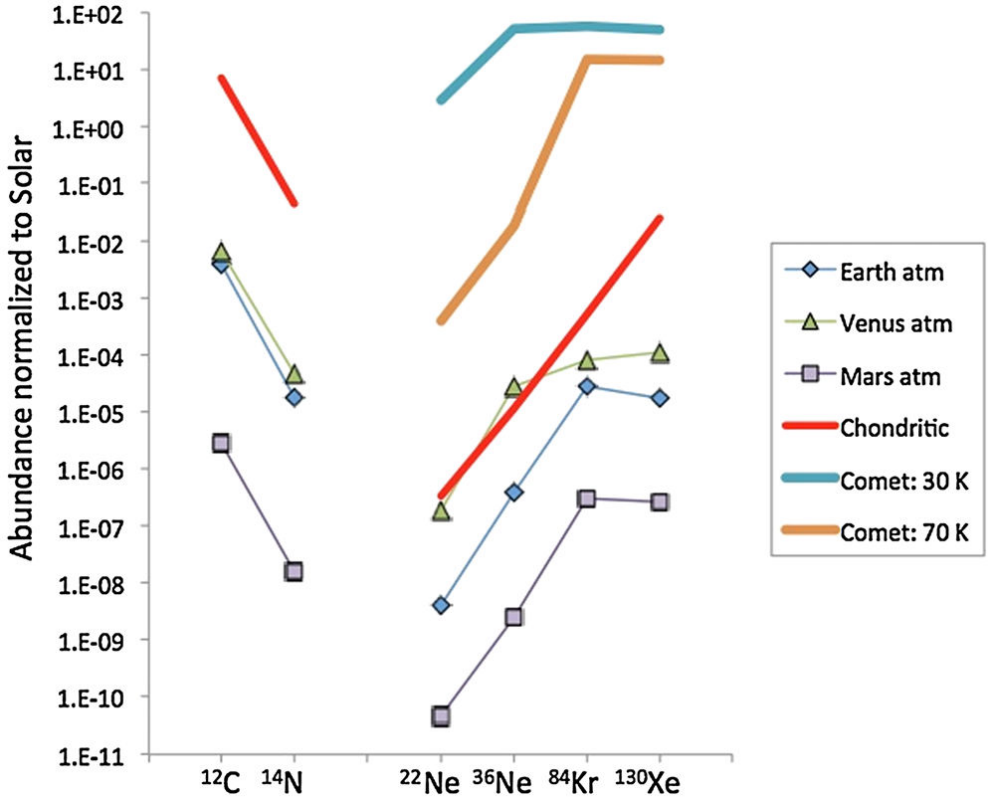
Volatile element abundances (mol/g) normalized to Solar (Lodders 2003) for terrestrial planet atmospheres compared to chondritic and cometary abundances. See Table 5 references for sources of data. Volatile abundances of inner planet atmospheres fit better with chondritic than presumed cometary patterns, keeping in mind that for comets’ real abundances are not yet measured. Heavy noble gases (e.g. Xe) appear nonetheless depleted relative to chondritic, indicating that a chondritic origin for planetary noble gases do not fully account for observed patterns (see the isotope subsection)

**Fig. 11:**
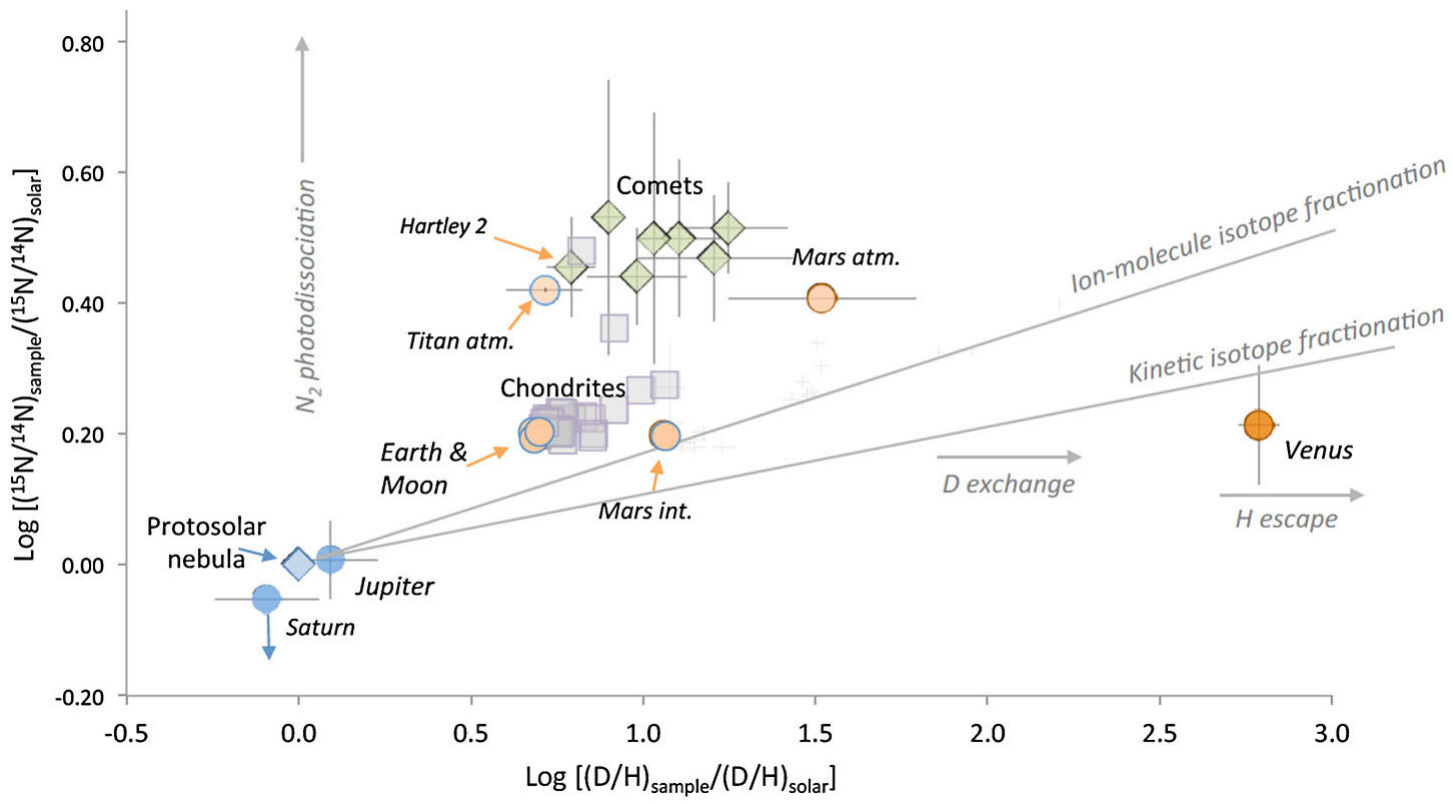
Co-variations of the H and N isotope ratios among solar system reservoirs and objects. The D/H and ^15^N/^14^N ratios are normalized to the PSN values ([2.1 ± 0.5] × 10^−5^ and [2.27 × 0.03] × 10^−3^, respectively; after Aléon 2010). Data are consistent with different processes of isotope fractionation, e.g., ion-molecule isotope fractionation during low temperature isotope exchange between organics and the protosolar gas, kinetic isotope fractionation proportional to the square root of mass, or self-shielding enhancement of the isotope fractionation through photodissociation of protosolar N_2_ by UV light (Clayton 2002; Chakraborty et al. 2013). The Earth shares H and N isotope signatures with bulk chondrites, whereas comets are richer in D and ^15^N, thus suggesting an asteroidal, rather than cometary, origin for terrestrial volatiles (Alexander et al. 2012; Marty 2012). The Venusian atmosphere is extremely depleted in water and rich in D as a consequence of photodissociation of H_2_O and subsequent loss (Grinspoon 1993). The enrichment in both D and ^15^N of the Martian atmosphere is attributed to atmospheric escape processes. Sources—Moon: Füri et al. (2014), Kerridge et al. (1991); Venus: Grinspoon (1993), Hoffman et al. (1980); Mars: Leshin et al. (2013), Leshin (2000); chondrites: Deloule et al. (1998), Kerridge (1985), Robert (2003); Saturn: Fletcher et al. (2014), Macy and Smith (1978); Jupiter: Mahaffy et al. (1998), Owen et al. (2001); Titan atmosphere: Niemann et al. (2010); comets: Bockelée-Morvan et al. (2008); Ceccarelli et al. (2014), Hartogh et al. (2011a), Manfroid et al. (2009), Rousselot et al. (2014)

The elemental abundances of volatile elements (C, N, O and noble gases) elements and key isotope ratios for inner planet atmospheres, carbonaceous chondrites (thought to represent wet asteroids) and comets are given in Table 5 and displayed in Figs. 10 and 11.

The abundances of volatile elements are reasonably well known in the Sun, our best representative for the PSN, as the Sun concentrates more than 99 % of the solar system mass at present. Carbonaceous chondrites are presumably representative of volatiles in wet asteroids from the outer part of the asteroid belt. The latter are potential candidates for the contributors of volatile elements to inner planets, which require addition of oxidized material to make the present-day chemistries of Earth and Mars (e.g., oxygen fugacity; Wänke and Dreibus 1988). It has been long recognized that the abundance patterns of noble gases in the terrestrial planets (except He which is not retained in inner planet atmospheres) differ from the solar abundance and resemble that of primitive meteorites (see Fig. 9). For this reason, these abundance patterns have been named “planetary” (e.g., Mazor et al. 1970) in contrast to “solar”. This denomination is, however, misleading to some point since some of the key isotope ratios of planetary atmospheres (for instance those of Xe) differ significantly from those of primitive meteorites.

Nevertheless, the abundance ratios of noble gases, and by extension of C and N in the atmospheres of Earth, Venus and Mars are quite consistent each other and resemble indeed those of carbonaceous chondrites (Fig. 10). A notable exception is Xe, which is underabundant in inner planet atmospheres by one order of magnitude relative to chondritic and other noble gases (e.g., Kr), an important difference to which we shall return later.

However, it is difficult to evaluate the possibility of a cometary origin based on noble gases simply because the composition of noble gases in comets has not yet been measured. Attempts to measure noble gases by spectroscopy have failed, and it is hoped that mass spectrometers onboard the Rosetta spacecraft will have the sensitivity to detect and, hopefully, quantify noble gases (Balsiger et al. 2007). In the absence of real data, it has been proposed that noble gases in comets could have been trapped from the PSN gas during the growth of amorphous ice, so that results of experiments aimed at measuring the trapping efficiency of noble gases in amorphous ice (Bar-Nun and Owen 1998; Bar-Nun et al. 2007) have been used to estimate the noble gas content of comets (Dauphas 2003; Owen and Barnun 1995).

The trapping efficiency of noble gases in ice depends on ice formation temperature and on the noble gas mass, and in Fig. 10 we represent the cometary abundance patterns for two end-member temperatures of comet formation, using data from Bar-Nun and Owen (1998), Bar-Nun et al. (2007) and Dauphas (2003). In such a format, the absolute abundances are not as important as the relative ones, and one could conceivably find trapping conditions able to satisfy the planetary abundance pattern. Notably, a dual origin of the terrestrial atmosphere has been proposed by Dauphas (2003) in which the Earth first had a nebular gas atmosphere which was fractionated during escape, and then received its load of Xe-depleted cometary gases, explaining in this way the Xe deficiency of planetary atmospheres.

### 5.2 Asteroidal Versus Cometary Origin for Inner Planet Volatiles: H and N Isotopes

We know a little bit more about the isotopic composition of comets, thanks to spectroscopic measurements of the D/H and N isotopes (Bockelée-Morvan et al. 2008; Ceccarelli et al. 2014; Hartogh et al. 2011a; Manfroid et al. 2009; Rousselot et al. 2014; Shinnaka et al. 2014; Altwegg et al. 2015). As described in Sect. 2, the D/H and ^14^N/^15^N ratios display dramatic variations among solar system objects and reservoirs, thus allowing one to derive information on origins and relationships between reservoirs (Aléon 2010; Marty 2012; Marty et al. 2011). Figure 11 represents the co-variations of H and N isotope ratios for planetary atmospheres, comets, and meteorites normalized to the PSN values (see Fig. 11 caption for data sources). All reservoirs but the giant planets are enriched in D and ^15^N compared to the PSN.

The causes of such enrichments are likely to result from extensive isotope fractionation during either ion-molecule reactions at low temperature, isotope exchange with D-rich molecules for H, isotope-selective photolysis, self shielding, or a combination of several of these processes. Notably, the Earth, the Moon and the interior of Mars and Venus all have comparable ^15^N/^14^N ratios that are also within the range of most values seen in primitive meteorites. The atmospheres of Titan and of Mars are exceptions (Mandt et al. 2015): for the Titan atmosphere, the enrichment in ^15^N in N_2_ (Niemann et al. 2010) is attributed to contribution of ^15^N-rich material akin of cometary amines and nitrides (Mandt et al. 2014), whereas the enrichment in ^15^N of the atmosphere of Mars is regarded as resulting from isotope-selective non-thermal escape processes (e.g., Bogard et al. 2001). In contrast to inner solar system bodies, all comets analyzed so far present strong enrichments (factors of about 2) in ^15^N in HCN (Bockelée-Morvan et al. 2008; Manfroid et al. 2009) and NH_3_ (Rousselot et al. 2014; Shinnaka et al. 2014). Although N_2_ has been detected by ROSINA DFMS in 67P/CG, its abundance was found to be depleted by a factor of ~25.4 ± 8.9 relative to CO (Rubin et al. 2015) and no isotope ratio has been measured. It is unclear if the depletion of N_2_ in comets is primordial or due to loss after accretion (Mousis et al. 2012), so it is difficult to conclude based on this early measurement if comets contributed significantly to the Earth’s nitrogen.

A similar, but somewhat more blurred, situation arises from D/H systematics. The Earth and the Moon and many meteorites share a common range of values about a factor of 6 enriched in D relative to the PSN. Venus is extremely rich in D, suggesting water loss through photodissociation of H_2_O and isotope-selective hydrogen escape (Grinspoon 1993). The Martian atmosphere is also rich in D when compared to Earth and chondrites, an enrichment equally attributed to selective (charge-related) atmospheric escape as in the case of N (ibid.). Thus it appears that, based on these isotope H and N variations, asteroids fit better than comets for the origin of inner planet volatiles.

Recently, terrestrial-like D/H ratios have been reported for two comets presumed to be JFCs (Ceccarelli et al. 2014; Hartogh et al. 2011a), thus revitalizing the theory for a cometary origin for terrestrial oceans. However, the measurement of the D/H ratio of comet 67P/CG counters this argument (Altwegg et al. 2015). It is important to note that comet Hartley 2, for which an ocean-like D/H ratio was measured (Hartogh et al. 2011a), is nevertheless rich in ^15^N compared to Earth, thus contradicting a cometary origin for terrestrial volatiles. The story may, however, evolve because we may not know fully the N isotope composition of comets. Indeed only CN, HCN and NH_2_ (presumably from ammonia) could be analyzed by spectroscopy, and it is possible that comets could also contain N^2^ from the PSN. If so, mixing of ^15^N-poor PSN nitrogen with ^15^N-rich nitriles and amines could conceivably yield inner solar system-like N isotope compositions, provided a good mix in the cocktail of 1/3–2/3 approximately.

### 5.3 Asteroidal Versus Cometary Origin for Inner Planet Volatiles: Other Isotope Systems

Neon has three isotopes—^20^Ne, ^21^Ne, ^22^Ne—which show significantly large variations in the solar system between a PSN ^20^Ne/^22^Ne end-member of 13.8 for the solar wind (possibly 13.4 for the PSN; Heber et al. 2012) and about 8.5 for the carbonaceous chondrite end-member (i.e., Ne-A; Mazor et al. 1970).

**Fig. 12:**
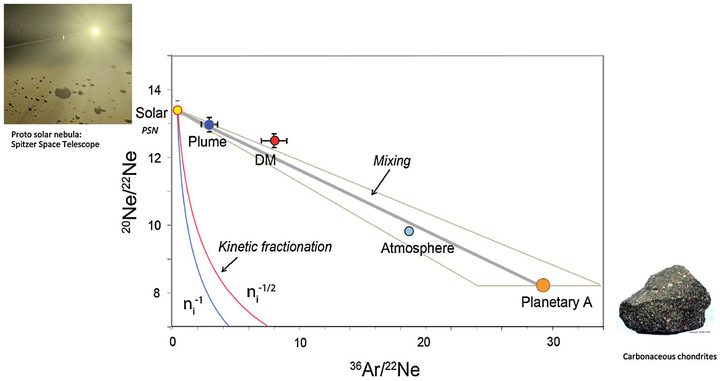
Co-variations of the ^20^Ne/^22^Ne isotopic ratio vs. the ^36^Ar/^22^Ne ratio for terrestrial reservoirs compared to the PSN (Heber et al. 2012) and the carbonaceous chondrite (Planetary-A component as defined by Mazor et al. 1970) end-members. In this format, two end-member mixing results in a straight line connecting the end-member data points. The terrestrial shallow depleted mantle (DM—Moreira et al. 1998), the deep mantle, represented by mantle plume (plume) material (Mukhopadhyay 2012; Yokochi and Marty 2004), and the atmosphere fit remarkably well a mixing relationship between a PSN-like component, presumably retained deep in the Earth’s interior, and a surficial reservoir—the atmosphere sensu largo—that was contributed by a wet asteroid end-member of carbonaceous chondrite composition. This diagram also illustrates the fact that the terrestrial atmosphere did not experience isotope-element fractionating escape processes, represented here by mass-dependent trajectories (*blue* and *red curves*). If atmospheric escape took place, it should have been non-fractionating, of the blown-off type (adapted from Marty 2012)

Solar-like neon has been identified in the terrestrial mantle (Mukhopadhyay 2012; Yokochi and Marty 2004), suggesting that the proto-Earth grew up in the presence of PSN gas, possibly under the form of a PSN, H_2_-rich atmosphere that equilibrated with molten silicates of a magma ocean. Later on, this primary atmosphere would have been blown off and a secondary atmosphere would have developed from contribution of wet bodies. Ne isotope and Ar-Ne abundances fit very well into this scenario and further indicate carbonaceous chondrite-type material as the best progenitor for the secondary atmosphere (and oceans—Fig. 12). The important relationship depicted in Fig. 12 indicates that terrestrial noble gases are fully consistent with mixing between a deep component of PSN origin and a more surficial component, mostly in the atmosphere, that was contributed by material having a composition akin of carbonaceous chondrites, therefore presumably wet asteroidal matter.

A cometary contribution would not fit well into this scheme, but cannot be totally excluded since one does not know the noble gas composition of comets. The only measurement of noble gases in cometary material is from the analysis of grains returned by the Stardust mission (Brownlee et al. 2006), which showed the occurrence of extraterrestrial He and Ne (Marty et al. 2008). Only fragments of one grain that exploded in the collecting medium (aerogel) could be analyzed so far, which yielded a ^3^He/^4^He ratio intermediate between the post-deuterium burning composition of the solar wind, and that of the Jupiter, probably representing the PSN composition. It is therefore possible that comets have taken snapshots of the early evolution of the Sun in the composition of noble gases they trapped at different periods of time.

The Stardust Ne isotopic composition is surprisingly different from the solar composition, and instead close to that of the terrestrial atmosphere. It is also indistinguishable, within errors, from the composition of Phase Q Ne, an ubiquitous component found in meteorites and thought to be associated with primitive organic matter (Marty et al. 2008). These unique results suggest that comets may also contain noble gases trapped in their organics and not only frozen in ice. Furthermore, the concentration of Ne in the Stardust material was found to be extremely large, of the order of 5 × 10^−7^ mol/g. With such concentration, the contribution of comets to atmospheric Ne could have been significant, with roughly the required ^20^Ne/^22^Ne ratio.

For the purpose of illustration, the terrestrial late heavy bombardment (TLHB) around 3.8 Ga ago (about 1 × 10^23^ g, as scaled to the lunar cratering record) could have been made of approximately 50 % Kuiper Belt material, according to the Nice model (Gomes et al. 2005). With such a concentration, about 5 × 10^16^ moles of neon could have been contributed by the TLHB, one order of magnitude more than the atmospheric inventory of neon (3.2 × 10^15^ moles). Marty and Meibom (2007) have argued that the TLHB could not be made of 50 % cometary material, based on the noble gas content of comets (estimated from amorphous ice experiments, see above). They proposed an upper limit of about 0.5 % for cometary matter in the TLHB, the rest consisting of asteroidal matter. With such contribution, about 10 % atmospheric Ne could have been contributed by cometary matter.

Another intriguing isotope system relevant to the present discussion is that of the nine isotopes of xenon, the heaviest stable noble gas. Besides several isotopes produced by extinct and extant radioactivities which have their own interest for geochronological purpose, the stable isotopes of xenon in the terrestrial atmosphere as well as in the Martian atmosphere (unfortunately, Venus is not measured) are mass-fractionated relative to either solar, or meteoritic Xe. The extent of fractionation is so large (3–4 % per amu) for such a heavy element that no known kinetic mass fractionation can account for this difference. Together with the xenon elemental depletion relative to the other noble gases, this unique isotope fractionation could in fact provide the signature of cometary matter that would carry an exotic Xe component from the edge of the solar system not seen in its inner region.

This possibility is not without problems, since it requires the existence of a component not seen elsewhere in the solar system, and which must be different from Xe in the Sun. Instead several models call for early processing of solar-like Xe in the terrestrial atmosphere. Pepin (1991, 2006) proposed that terrestrial Xe was mass fractionated from an initial composition resembling the solar one during episodes of magma ocean degassing and atmospheric escape. One problem with such a possibility is that it would require the same suite of events occurring on Mars (whose atmosphere is equally fractionated for Xe isotopes), which is difficult to conceive for such drastically different planets.

Dauphas (2003) proposed a dual origin for the terrestrial atmosphere, in which a solar composition was fractionated during escape, leaving only isotopically fractionated xenon, and then contributed to by cometary gases depleted in xenon, leaving thus the fractionated Xe signature while providing the isotope compositions of the other noble gases. This attractive model may also suffer from the comparison with Mars—that is, requiring a similar suite of events for a planet very different from the Earth in terms of heliocentric distance and size.

More recently, Pujol et al. (2011) found that xenon trapped in Archean (3.5 Ga-old) sedimentary rocks is isotopically intermediate between chondritic/solar, and modern atmospheric Xe. They attributed this composition to the signature of the ancient atmosphere at that time. They proposed that the Xe isotope fractionation and Xe underabundance was due to an atmospheric escape process specific to Xe and not affecting other noble gases. This escape was progressive through time, and was not yet completed 3.5 Ga ago. The specific Xe atmospheric escape would be related to its electronic structure together with a higher far UV light flux in the distant past that would have enhanced selective loss of xenon from the atmosphere through time. If this were the case, the xenon “paradox” would no longer be a question of the nature of the extraterrestrial source (cometary versus asteroidal), but an aeronomic problem specific to Earth and Mars. All these models cannot be fully tested at present because we lack precise measurements of noble gases in comets.

### 5.4 Conclusion

With all of the data and information at hand, it appears that contribution from asteroidal material of the type of that sampled by carbonaceous chondrites can better explain the composition of the inner planet atmospheres than contribution of cometary matter. This appears to be the case for the abundances of noble gases, and for the isotopes of hydrogen and nitrogen. However, the situation is severely biased by the lack of cometary data: no heavy noble gases have been measured so far in these objects, and measurements of elemental and stable isotope compositions have considerable uncertainties that are inherent to the spectroscopic methods. Many of the considerations above are based on a very restricted number of data and on considerable assumptions, and they certainly highlight the need to have more data on the volatile content and isotopic composition of cometary volatiles.

## 6 Pluto and Kuiper Belt Objects

Pluto’s mean density shows that it is poor in ice and suggests that it formed in a water-poor region of the solar nebula (Lunine 1989). It is also likely to have experienced further loss of volatiles by the impact formation of Charon (McKinnon and Mueller 1988). The surface of Pluto consists of a spatially heterogeneous mixture of N_2_, CH_4_, CO and C_2_H_6_ ices (Cruikshank et al. 2015). Molecular nitrogen is the most abundant ice on the surface and is presumed to be the primary constituent in Pluto’s tenuous atmosphere (Owen et al. 1993) where CH_4_ and CO have been detected (Lellouch et al. 2011). N_2_ ices have also been detected on the surface of several Kuiper Belt objects, including Sedna (Barucci et al. 2010) and Quaoar (Dalle Ore et al. 2009).

The source of nitrogen for the surface ices and atmosphere of Pluto can provide important information about its formation conditions, particularly about the temperature and composition of the region of the PSN in which it formed. Like Triton, Pluto’s nitrogen originated either as N_2_ or NH_3_ in the PSN. The abundance of N_2_ is believed to have been greater in the PSN than NH_3_ (Lewis and Prinn 1980), but would only have been accreted by Pluto if it formed at temperatures less than 38 K (Bar-Nun et al. 1985, 1988).

The formation conditions of comets provide important information relevant to the formation of Pluto. Based on measurements currently available, comets are found in general to be deficient in N_2_ relative to NH_3_. This means that temperature conditions in the region where comets (and Pluto) formed could have been too warm for N_2_ ice to form, that they did not retain N_2_ beyond their first pass through the solar system (Owen et al. 1993), or that detection methods for N_2_ are too limited to determine its abundance in comets. The recent detection of N_2_ in 67P/CG has been interpreted to mean that this comet formed at temperatures less than 30 K (Rubin et al. 2015), although the N_2_/CO ratio is compatible with formation temperatures as high as 56 K if the nucleus agglomerated from ice grains made of clathrates (Mousis et al. 2012; Rubin et al. 2015). As with Titan (Mandt et al. 2014), if formation temperatures were low enough the source of Pluto’s nitrogen was N_2_ in the PSN, but if temperatures were higher than 38 K then the source of nitrogen for Pluto’s surface and atmosphere would have been NH_3_ in the PSN that was somehow converted to N_2_. Nitrogen isotope ratios could provide important constraints on the origin on Pluto’s nitrogen if the escape history of Pluto’s atmosphere can be constrained as has been done with Titan (Mandt et al. 2014). Determining the origin of Pluto’s nitrogen would have important implications for understanding the origin of Kuiper Belt objects and Neptune’s moon Triton.

## 7 Discussion and Conclusions

We review here the state of knowledge on the formation of the planets, moons and small bodies in the solar system and the contributions that comets have made to this understanding. There are many limitations to this understanding, so we also highlight measurements that are needed to address these limitations. The European Space Agency (ESA) Rosetta mission (Glassmeier et al. 2007) is currently in orbit around comet 67P/CG and the NASA New Horizons spacecraft (Stern 2009) will fly by Pluto in July 2015. Both of these missions will take measurements that will help address these limitations as well as the upcoming ESA JUpiter ICy moons Explorer (JUICE) mission and a NASA Europa mission.

### 7.1 Current State of Knowledge and Limitations

The composition of giant planet atmospheres demonstrates that heavy elements are enriched in the giant planets. This is interpreted to mean that the building blocks of the giant planets were a mixture of rocks and crystalline ices (e.g. Gautier et al. 2001). The exact nature of these ices is still not determined, although most recent results seem to argue for full clathration of volatiles to explain the enrichment of volatiles in the giant planet atmospheres and the ^14^N/^15^N in Jupiter’s (Mousis et al. 2009c, 2012) and Saturn’s atmospheres (Mousis et al. 2014a). The formation scenarios of Uranus and Neptune are not as well constrained and could also be explained by formation in a region between the CO and N_2_ ice lines (Ali-Dib et al. 2014). Questions remain as to the later contribution of comets to the volatile inventories of the giant planet atmospheres. The example of the SL9 impact with Jupiter demonstrated that CO and HCN produced by shock chemistry remain in a giant planet atmosphere long after a comet impact and can be used as tracers for recent comet contributions to the volatile inventory of giant planets.

The giant planet satellites are divided into two categories based on their origin. Captured satellites could be similar in composition to comets because they are likely to have formed in conditions similar to the formation conditions of Kuiper Belt and Oort Cloud objects while regular satellites formed as a byproduct of giant planet formation. However, the fact that comets appear to be deficient in nitrogen while Pluto and Triton, both presumed to be Kuiper Belt objects, contain significant nitrogen is a puzzle yet to be resolved. Current understanding of regular satellites is that they formed in a gas-starved disk around their planet and that they are expected to have large inventories of water ice and other volatiles (Canup and Ward 2002, 2006). Their composition is an important tool for providing further constraints on their origin. The D/H ratio in water in Enceladus demonstrates that the building blocks for Saturn’s regular satellites must have formed in the PSN where conditions were cold enough to provide a D/H similar to that of OCCs and to trap N_2_, CO and ^36^Ar (Mousis et al. 2009b). The deficiency of CO and ^36^Ar in Titan’s atmosphere, as well as a ^14^N/^15^N in N_2_ that indicates its origin as NH_3_ in the PSN suggests that the building blocks for Saturn’s regular satellites partially devolatilized prior to formation of the moons (Mousis et al. 2009a; Mandt et al. 2014). Knowledge about the composition of Jupiter’s moons is limited primarily to their ice-to-rock ratio. This parameter is either an indication of their formation distance in Jupiter’s subnebula (e.g. Canup and Ward 2002; Mousis and Gautier 2004) or they initially had high ice-to-rock ratios, and have lost part or all of their ice inventory as a result of tidal heating (Estrada et al. 2009). Due to extensive thermal processing and radiolitic chemistry on the surface, it is difficult to provide any further constraints on the origin of these moons based on what is currently known about their composition.

Even less is known about the moons of Uranus and Neptune beyond the fact that Triton is a captured moon with extensive surface ices composed primarily of N_2_ with trace amounts of CH_4_ and CO. This observation combined with similar observations of the atmosphere and surface composition of Pluto suggest that Kuiper Belt objects may have formed in conditions cold enough to trap and retain N_2_ and CO, but more data are needed to confirm this.

The data available to evaluate volatile origins of the terrestrial planets point toward asteroidal material of the type of that sampled by carbonaceous chondrites to explain the composition of the inner planet atmospheres as opposed to contribution of cometary matter. This agrees with the abundances of noble gases, as well as the isotopes of hydrogen and nitrogen.

### 7.2 Measurements that Are Needed

Now that we have reviewed measurements that have implications for understanding the formation and evolution of the solar system, areas where more measurements are needed clearly stand out.

First, there are only ten measurements of D/H in water in comets currently available. Two of the three JFCs that were measured have D/H values in water in the range of D/H in water for Earth, while the D/H in water for 67P/CG and seven OCCs is a factor of 2–4 greater than the terrestrial ratio. This statistical sampling is far too low for any significant conclusions to be made. Further measurements of D/H in comets is desperately needed to enhance understanding of solar system formation processes and the role of comets in contributing volatiles to planetary atmospheres. The OPR measurements show trends that are also relevant, but only a few limits and three measurements are available for JFCs. More information about JFCs and more measurements in OCCs are needed.

Future work on carbon isotope ratios should include expanding number of C_2_ measurements and inclusion of other carbon bearing species, particularly among the photochemical parents of the more commonly studied visible radicals. Combined with increasing the measurement and modeling precision of the existing methods and narrowing the selection of potential parents it will be possible to determine if there are any potential trends with heliocentric distance and to evaluate if a subtle shift toward higher values in comets relative to Earth is significant. Future work on nitrogen isotope ratios will require the acquisition of new HCN radio measurements in coordination with NUV CN, and expanding measurements of ^14^N/^15^N in cometary NH_3_.

The oxygen and sulfur isotopic measurements in comets are not sufficient to provide any assistance in understanding formation and evolution of the solar system. Although the general trend for cometary ^16^O/^18^O ratios is consistent with the range described between chondritic material and asteroidal water, more effective classification will require not only more measurements, but also more precise ones that include ^17^O. A larger sample size of sulfur isotope ratios is needed that targets multiple isotopes from several comets of different classes and heliocentric distances in order for these ratios to be compared to other solar system measurements.

Noble gas measurements have only been made in the atmospheres of Jupiter, Titan and the terrestrial planets. In order to better understand the formation processes in the solar system, the noble gas abundances need to be measured in several comets as well as in the atmospheres of Saturn, Uranus and Neptune and other satellites of giant planets.

To continue evaluation of the contribution of comets to giant planet atmospheric composition, further observations of CO and HCN in giant planet atmospheres are needed. This continuing study will not only benefit the Solar System atmosphere field but also the growing field of exoplanet atmosphere characterization (Turrini et al. 2014).

The origin of Kuiper Belt objects can be constrained through nitrogen isotope ratio measurements. Measuring the ^14^N/^15^N in the atmospheres of Triton, Pluto and several Kuiper Belt objects would significantly improve understanding of their formation conditions. A very high isotope ratio would suggest origin in very cold conditions, while a very low ratio would indicate origin as NH_3_ (assuming that escape and photochemistry have not significantly changed the ratio over time).

Evaluation of the terrestrial planet atmospheres is severely biased by the lack of cometary data: no heavy noble gases have been measured so far in these objects, and measurements on elemental and stable isotope compositions have considerable uncertainties that are inherent to the spectroscopic methods. The conclusions made here are based on a very restricted number of data and on considerable assumptions, and they certainly highlight the need to have more data on the volatile content and isotopic composition of cometary volatiles.

### 7.3 Expected Contributions of Rosetta, New Horizons and Other Upcoming Missions

The Rosetta mission has now measured the D/H in another JFC and shown that the trend of a different D/H in different comet families is complicated. D/H ratios in CH_4_ and HCN and ^14^N/^15^N in HCN and NH_3_ are also anticipated along with the noble gas abundances. These measurements will not only add to the statistics on comets but also provide new measurements that may change perspectives on solar system formation. This mission is of high value and more missions like Rosetta are needed in the future.

The atmosphere of Saturn will continue to be studied by the Cassini mission. When this mission reaches the end of its operational life, the spacecraft will deorbit into the atmosphere of Saturn. This will provide a prime opportunity to measure *in situ* the noble gas abundances in Saturn’s atmosphere as well as isotope ratios of some of the major atmospheric constituents.

The JUICE mission and an upcoming NASA-sponsored Europa mission may be able to provide improved composition measurements for the Galilean moons that could include isotopic composition, while the ALICE UV spectrometer (Stern et al. 2008) on the New Horizons mission may be able to measure the ^14^N/^15^N in Pluto’s atmosphere if the ratio is ≤ 330 (Jessup et al. 2013).

Although much has been learned by observations of comets and solar system bodies, a great deal of work remains.

## Figures and Tables

**Table 1:** Elemental abundances in the Sun and protosun (adapted from Lodders et al. 2009)

Element	Solar dex	Protosolar dex	Δ dex	Protosolar X/H	Protosolar X/H_2_	Δ (X/H_2_)
He	10.93	10.99	0.02	9.68 × 10^−2^	1.94 × 10^−1^	9.13 × 10^−3^
C	8.39	8.44	0.04	2.77 × 10^−4^	5.55 × 10^−4^	5.35 × 10^−5^
N	7.86	7.91	0.12	8.18 × 10^−5^	1.64 × 10^−4^	5.21 × 10^−5^
O	8.73	8.78	0.07	6.07 × 10^−4^	1.21 × 10^−3^	2.12 × 10^−4^
Ne	8.05	8.10	0.10	1.27 × 10^−4^	2.54 × 10^−4^	6.56 × 10^−5^
P	5.46	5.51	0.04	3.26 × 10^−7^	6.52 × 10^−7^	6.29 × 10^−8^
S	7.14	7.19	0.01	1.56 × 10^−5^	3.12 × 10^−5^	7.27 × 10^−7^
Ar	6.50	6.55	0.10	3.57 × 10^−6^	7.15 × 10^−6^	1.85 × 10^−6^
Kr	3.28	3.33	0.08	2.15 × 10^−9^	4.31 × 10^−9^	8.71 × 10^−10^
Xe	2.27	2.32	0.08	2.10 × 10^−10^	4.21 × 10^−10^	8.51 × 10^−11^

**Table 2:** Percent contributions of the isotopes of main volatile elements. These values are adapted from Lodders et al. (2009) with the exception of the nitrogen (from Marty et al. 2011) and carbon (from Hashizume et al. 2004)

Element	Atomic mass	Atom (%)
H	1	99.9981
H	2	0.00194
		100
He	3	0.0166
He	4	99.9834
		100
C	12	98.998
C	13	1.001
		100
N	14	99.774
N	15	0.226
		100
O	16	99.763
O	17	0.037
O	18	0.200
		100
Ne	20	92.9431
Ne	21	0.2228
Ne	22	6.8341
		100
P	31	100
S	32	95.018
S	33	0.75
S	34	4.215
S	36	0.017
		100
Ar	36	84.595
Ar	38	15.381
Ar	40	0.024
		100
Kr	78	0.362
Kr	80	2.326
Kr	82	11.655
Kr	83	11.546
Kr	84	56.903
Kr	86	17.208
		100
Xe	124	0.129
Xe	126	0.112
Xe	128	2.234
Xe	129	27.463
Xe	130	4.378
Xe	131	21.802
Xe	132	26.355
Xe	134	9.661
Xe	136	7.868
		100

**Table 3:** Elemental and isotopic abundances in the four giant planets

Element	Jupiter/Sun	Saturn/Sun	Uranus/Sun	Neptune/Sun
He	0.8^a^	0.7 ± 0.1^g^	0.9 ± 0.2^n^	1.2 ± 0.2^r^
Ne	0.1^b^	–	–	–
O	0.4 ± 0.1^c^	(1.6 ± 0.29) × 10^—4h^	–	–
C	4.3 ± 1.1^c^	9.6 ± 1.0^i^	41.5 ± 16.7^o^	72.1 ± 19.3^o^
N	4.1 ± 2.0^c^	2.8 ± 1.1^j^	–	–
S	2.9 ± 0.7^c^	12.05^k^	22.5 ± 11.3^p^	22.5 ± 11.3^p^
P	3.3 ± 0.4^d^	11.2 ± 1.3^d^	–	–
Ar	2.5 ± 0.8^b^	–	–	–
Kr	2.2 ± 0.6^b^	–	–	–
Xe	2.1 ± 0.6^b^	–	–	–
Isotope	Jupiter	Saturn	Uranus	Neptune
D/H (in H_2_)	(2.60 ± 0.7) × 10^−5e^	$1.70_{-0.45}^{+0.75}\times 10^{-51}$	(4.4 ± 0.4) × 10^—5q^	(4.1 ± 0.4) × 10^—5q^
^3^He/^4^He	(1.66 ± 0.05) × 10^−4e^	–	–	–
^12^C/^13^C (in CH_4_)	$92.6_{-4.1}^{+4.5}$^f^	$91.8_{-7.8}^{+8.4}$^i^	–	–
^14^N/^15^N (in NH_3_)	$434.8_{-50}^{+65}$^c^	> 500^m^	–	–

Error is defined as (ΔE/E)^2^ = (ΔX/X_planet_)^2^ + (ΔX/X_Protosun_)^2^.

a von Zahn et al. (1998);

b Mahaffy et al. (2000);

c Wong et al. (2004);

d Fletcher et al. (2009a);

e Niemann et al. (1998);

f Niemann et al. (1996);

g Conrath and Gautier (2000);

h de Graauw et al. (1997);

i Fletcher et al. (2009b);

j Fletcher et al. (2011a);

k Briggs and Sackett (1989);

l Lellouch et al. (2001);

m Fletcher et al. (2014);

n Conrath et al. (1987);

o Karkoschka and Tomasko (2009, 2011);

p de Pater et al. (1991);

q Feuchtgruber et al. (2013);

r Conrath et al. (1991).

**Table 4:** Measurements of Titan’s current and *primordial* composition compared to what has been measured in comets and determined for the PSN

		Titan	Comets	PSN
CO:CH_4_		~10^−3a^		70^b^
D/H	H_2_	1.35 ± 0.3 × 10^−4c^	–	1.94 × 10^−5d^
	H_2_O	$2.9_{-0.7}^{+1.5}\times 10^{-4}$^e^	1.5–5.3 × 10^−4f^	variable
	CH_4_	1.6 ± 0.3 × 10^−4g^ *0.95–1.6* × *10*^−*4*h^	–	variable
^14^N/^15^N	N_2_	167.7 ± 0.6^c^	–	430 ± 50^i^
	NH_3_	≤ *190*^j^	127±32^k^ 139 ± 38^l^	130±40^m^
	HCN	56 ± 8^n^	157 ± 21^o^	157 ± 21^o^
^22^Ne/N	Ne	1.4 ± 1.1 × 10^−7c^	–	0.106^d^
^36^Ar/N	Ar	1.1 ± 0.4 × 10^−7c^	–	3.67 × 10^−2d^
Kr/N	Kr	< 5 ×10^−9c^	–	2.63 × 10^−5d^
Xe/N	Xe	< 5 ×10^−9c^	–	2.58 × 10^−6d^

a Gautier and Raulin (1997);

b Lodders (2003);

c Niemann et al. (2010);

d Lodders et al. (2009);

e Waite et al. (2009);

f See Fig. 4;

g Nixon et al. (2012);

h Mandt et al. (2012);

i Based on solar wind from Marty et al. (2011) and Jupiter’s atmosphere from Owen et al. (2001);

j Mandt et al. (2014);

k Rousselot et al. (2014);

l Shinnaka et al. (2014);

m Based on comet measurements;

n Vinatier et al. (2007);

o Bockelée-Morvan et al. (2008).

**Table 5:** Elemental abundances of volatile elements and key isotope ratios in the atmospheres of Earth, Venus and Mars, compared to those of chondrites, of model cometary matter and of the PSN. The inner planet abundances are the surface inventories divided by the mass of the respective planets. The Earth atmosphere represents the sum of the oceans, sediments and atmosphere stricto sensu. Cometary abundances are those in amorphous ice for two end-member formation temperatures of 30 K and 70 K, respectively

Isotope	Earth atm.^a^	Venus atm.^a,b^	Mars atm.^a,c^	Chondritic^d^	Cometary @ 30 K^e^	Cometary @ 70 K^e^	PSN^f^
^12^C	1.29 × 10^−6^	2.17 × 10^−6^	9.25 × 10^−10^	2.28 × 10^−3^	–	–	3.25 × 10^−4^
14_N_	5.98 × 10^−8^	1.57 × 10^−7^	5.21 × 10^−11^	1.44 × 10^−4^	–	–	3.36 × 10^−3^
^1^H	1.48 × 10^−5^	–	–	6.14 × 10^−3^	5.40 × 10^−2^	5.40 × 10^−2^	–
^22^ Ne	2.27 × 10^−14^	1.07 × 10^−12^	2.58 × 10^−16^	1.89 × 10^−12^	1.62 × 10^−5^	2.28 × 10^−9^	5.74 × 10^−6^
^36^Ar	9.58 × 10^−13^	6.97 × 10^−11^	6.00 × 10^−15^	2.89 × 10^−11^	1.30 × 10^−4^	4.64 × 10^−8^	2.49 × 10^−6^
^84^Kr	1.98 × 10^−14^	5.60 × 10^−14^	2.10 × 10^−16^	3.61 × 10^−13^	3.92 × 10^−8^	1.03 × 10^−8^	6.95 × 10^−10^
^130^Xe	1.08 × 10^−16^	6.85 × 10^−16^	1.60 × 10^−18^	1.61 × 10^−13^	3.13 × 10^−10^	8.97 × 10^−11^	6.21 × 10^−12^
Isotope ratio							
D/H × 10^−4^	1.56	160 ± 20	8.6 ± 4.08^g^	1.7 ± 0.4	1.5–5.3^h^		0.21
14_N/_15_N_	272	270 ± 51	173 ± 11^g^	262 ± 29	130–160^i^		440^j^
^20^Ne/^22^Ne	9.80	–	~8(?)^g^	~8.5(Ne-A)	~10^k^		13.4–13.8^l^

a Pepin (1991);

b Hoffman et al. (1980);

c Bogard et al. (2001);

d from Orgueil and Murchison carbonaceous chondrite data in Marty (2012) and references therein;

e as computed by Dauphas (2003) with data from Bar-Nun and Owen (1998);

f Lodders et al. (2009);

g Garrison et al. (1998);

h Bockelée-Morvan et al. (2004); Hartogh et al. (2011a); and Altwegg et al. (2015);

i Rousselot et al. (2014) and Shinnaka et al. (2014);

j Marty et al. (2011);

k Marty et al. (2008) from Stardust cometary material;

l Grimberg et al. (2006)

## Abbreviations

AU: Astronomical Unit
CG: Churyumov-Gerasimenko
CIRS: Cassini Infrared Spectromer
dex: logarithmic abundance
DM: Depleted Mantle
ESA: European Space Agency
Ga: Billion years
GPMS: Galileo Probe Mass Spectrometer
HIFI: Heterodyne Instrument for the Far Infrared
HST: Hubble Space Telescope
INMS: Ion Neutral Mass Spectrometer
IRIS: Infrared Spectrometer
ISO: Infrared Space Observatory
JCMT: James Clerk Maxwell Telescope
JFC: Jupiter Family Comet
JUICE: JUpiter ICy moons Explorer
NRAO: National Radio Astronomy Observatory
NUV: Near Ultraviolet
OCC: Oort Cloud Comet
OPR: Ortho-to-Para Ratio
ppb: parts per billion
ppm: parts per million
PSN: Protosolar Nebula
SIS: superconductor insulator superconductor
STIS: Space Telescope Imaging Spectrograph
SWAS: Submillimeter Wave Astronomy Satellite
TLHB: Terrestrial Late Heavy Bombardment
UV: Ultraviolet
VSMOW: Vienna Standard Mean Ocean Water
